# Assembly of a heptameric STRIPAK complex is required for coordination of light-dependent multicellular fungal development with secondary metabolism in *Aspergillus nidulans*

**DOI:** 10.1371/journal.pgen.1008053

**Published:** 2019-03-18

**Authors:** Nadia Elramli, Betim Karahoda, Özlem Sarikaya-Bayram, Dean Frawley, Mevlüt Ulas, C. Elizabeth Oakley, Berl R. Oakley, Stephan Seiler, Özgür Bayram

**Affiliations:** 1 Biology Department, Maynooth University, Maynooth, Co. Kildare, Ireland; 2 Department of Molecular Biosciences, University of Kansas, 1200 Sunnyside Avenue, Lawrence, Kansas, United States of America; 3 Institute for Biology II—Molecular Plant Physiology, Albert-Ludwigs University Freiburg, Freiburg, Germany; 4 Maynooth University Human Health Research Institute, Maynooth, Co. Kildare, Ireland; Oregon State University, UNITED STATES

## Abstract

Eukaryotic striatin forms striatin-interacting phosphatase and kinase (STRIPAK) complexes that control many cellular processes including development, cellular transport, signal transduction, stem cell differentiation and cardiac functions. However, detailed knowledge of complex assembly and its roles in stress responses are currently poorly understood. Here, we discovered six striatin (StrA) interacting proteins (Sips), which form a heptameric complex in the filamentous fungus *Aspergillus nidulans*. The complex consists of the striatin scaffold StrA, the Mob3-type kinase coactivator SipA, the SIKE-like protein SipB, the STRIP1/2 homolog SipC, the SLMAP-related protein SipD and the catalytic and regulatory phosphatase 2A subunits SipE (PpgA), and SipF, respectively. Single and double deletions of the complex components result in loss of multicellular light-dependent fungal development, secondary metabolite production (e.g. mycotoxin Sterigmatocystin) and reduced stress responses. *sipA* (Mob3) deletion is epistatic to *strA* deletion by supressing all the defects caused by the lack of striatin. The STRIPAK complex, which is established during vegetative growth and maintained during the early hours of light and dark development, is mainly formed on the nuclear envelope in the presence of the scaffold StrA. The loss of the scaffold revealed three STRIPAK subcomplexes: (I) SipA only interacts with StrA, (II) SipB-SipD is found as a heterodimer, (III) SipC, SipE and SipF exist as a heterotrimeric complex. The STRIPAK complex is required for proper expression of the heterotrimeric VeA-VelB-LaeA complex which coordinates fungal development and secondary metabolism. Furthermore, the STRIPAK complex modulates two important MAPK pathways by promoting phosphorylation of MpkB and restricting nuclear shuttling of MpkC in the absence of stress conditions. SipB in *A*. *nidulans* is similar to human suppressor of IKK-ε(SIKE) protein which supresses antiviral responses in mammals, while velvet family proteins show strong similarity to mammalian proinflammatory NF-KB proteins. The presence of these proteins in *A*. *nidulans* further strengthens the hypothesis that mammals and fungi use similar proteins for their immune response and secondary metabolite production, respectively.

## Author summary

The multisubunit STRIPAK complex has been studied from yeast to human and plays a range of roles from cell-cycle arrest, fruit body formation to neuronal functions. Molecular assembly of the STRIPAK complex and its roles in stress responses are not well-documented. Fungi, with an estimated 1.5 million members are friends and foes of mankind, acting as pathogens, natural product and enzyme producers. In filamentous fungus *Aspergillus nidulans*, we found a heptameric STRIPAK core complex made from three subcomplexes, which sits on the nuclear envelope and coordinates signal influx for light-dependent fungal development, secondary metabolism and stress responses. STRIPAK complex controls activities of two major Mitogen Activated Protein Kinase (MAPK) signaling pathways through either promoting their phosphorylation or limiting their nuclear localization under resting conditions. These findings establish a basis for how fungi govern signal influx by using multimeric scaffold protein complexes on the nuclear envelope to control different downstream pathways.

## Introduction

Signaling pathways that regulate morphological and physiological processes in response to stimuli are often highly conserved throughout eukaryotes, signifying their importance. Striatin is one of the regulatory proteins proposed to act as a signalling hub for the control of many cellular processes including development, cellular transport and signal transduction [1, 2]. It forms a scaffolding platform to build the striatin-interacting phosphatase and kinase (STRIPAK) complex which is a large multimeric protein complex highly conserved in eukaryotes [3]. The STRIPAK complex influences mammalian cell size, morphology and migration [1]. It also plays a role in the polarisation of the golgi apparatus and is implicated in the process of mitosis through tethering vesicles of the golgi to the nuclear membrane and centrosomes [4].

The mammalian STRIPAK complex consists of a multitude of core members which include (i) Striatins (ii) Striatin-interacting proteins (STRIP1/STRIP2), (iii) monopolar spindle one-binder (Mob3/phocein) protein, [5], (iv) cerebral cavernous malformation 3 protein, CCM3 (v) and the phosphatase 2A subunits PP2AA and PP2Ac that have structural and catalytic functions, respectively. Further associated proteins include cortactin-binding proteins (CTTNBP), suppressor of IKKε (SIKE) and sarcolemmal membrane associated protein (SLMAP) and multiple germinal centre kinases (GCKIII), such as STK24, STK25 and MST4 that belong to the STE20 kinase family [3, 6]. The GCKs were discovered to be involved in the control of the cell cycle, polarity and migration [7, 8] and their functionality is reliant on CCM3 which is involved in stabilising the kinases [9].

The STRIPAK complex in the fruit fly *Drosophila melanogaster* acts as a negative regulator of the Hippo signaling pathway [10]. In *Saccharomyces cerevisiae*, the homologous complex is termed the Far complex which is implicated in cell cycle arrest and acts as an antagonist towards target of rapamycin complex 2 (TORC2) signaling [11, 12]. In the fission yeast *Schizosaccharomyces pombe*, the STRIPAK complex is known as the SIP complex (Septation initiation network [SIN] Inhibitory PP2A complex). This complex is necessary for coordinating mitosis and cytokinesis in *S*. *pombe* [13].

In closely related fungi *Neurospora crassa* and *Sordaria macrospora*, the STRIPAK complex controls cell-cell recognition, cell fusion and fruit body formation [6, 14, 15]. In *S*. *macrospora*, STRIPAK complex was initially discovered to be composed of PRO22 (STRIP1/2), PRO11 (STRN), PP2AA and PP2Ac1 by using Tandem Affinity Purification (TAP) coupled to mass spectrometry (MS) [14]. The SLMAP homolog PRO45 was also shown to be part of the complex [16]. The *N*. *crassa* STRIPAK complex, which is required for aerial mycelium formation, conidiospore formation and cell-cell signaling, is composed of the core HAM2 (STRIP), HAM3 (STRN), catalytic subunit PPG1 (PP2Ac) components and the accessory proteins HAM4 (SLMAP) and MOB3 (Mob3) [15]. In both fungi, the STRIPAK complex is made of STRIP, STRN, PP2AA, PP2Ac1, SLMAP and several GCKs [16]. It has very recently been shown that a SIKE homolog of *S*. *macrospora*, STRIPAK complex interactor (SCI1), a small coiled-coil protein, interacts with striatin (PRO11) and is required for hyphal fusion and fruit body development [17]. In both fungi, several subunits of the STRIPAK complex are associated with the nuclear envelope [15–17]. Furthermore, *N*. *crassa* STRIPAK is required for nuclear accumulation of mitogen activated protein kinase (MAPK), MAK-1, which is important for the cell-wall stress pathways as well as for cell-cell communication [15].

Fungi produce secondary metabolites (SM) ranging from beneficial antimicrobials, insecticides and antitumor agents to deleterious mycotoxins. SM genes, which are usually clustered, are expressed in response to environmental signals such as light, starvation and stress conditions [18, 19]. A model fungus frequently used to study SM production is *Aspergillus nidulans*. This fungus reproduces by forming asexual (conidia) or sexual (ascospores) spores [20, 21]. Germination of either type of spores leads to long hyphal filaments that in response to different light regimes develop into either asexual or sexual organs. In the light, the hyphae mainly form aerial conidiophores that produce clonal conidia. In the dark, the fungus switches to sexual reproduction and formation of ascospores that are formed inside of multicellular higher-ordered spherical fruit bodies (cleistothecia). Defects in the different developmental programs frequently impair SM production [20]. *A*. *nidulans* produces many metabolites, including the carcinogenic mycotoxin Sterigmatocystin (ST), antibiotic Penicillin (PN) and antitumour agent Terrequinone (TQ) [22]. Development and SM production are controlled by regulatory complexes, including the light-operated heterotrimeric VeA-VelB-LaeA complex. The velvet complex is formed in the nucleus where it controls gene expression required for sexual development and SM production [23]. The velvet complex is further controlled by the MAPK MpkB (yeast Fus3 ortholog), which phosphorylates VeA and is involved in regulation of cell-cell fusion, sexual development and SM production. All upstream kinases of MpkB, like MAP3K SteC (yeast Ste11p), MAP2K MkkB (yeast Ste7p) and the adaptor protein SteD (yeast Ste50p) also participate in sexual development and SM production [24, 25].

In *A*. *nidulans*, striatin (StrA) is localized to the nuclear envelope and loss of StrA results in pleitropic effects, including reduced hyphal growth, conidiation and loss of ascospores [26]. However, the molecular composition of the *An*STRIPAK complex, where it localizes, how it controls growth and development and whether it is involved in SM production are currently unknown. Therefore, we have attempted to understand mechanistic functions of the *An*STRIPAK complex. We report the composition of the *An*STRIPAK complex, its subcellular assembly and its roles in fungal development and SM production. Furthermore, we show the presence of SIKE-like protein as a component of the *An*STRIPAK in fungi in addition to *S*. *macrospora* coiled-coil domain protein SCI1 [17] and present the functional interplay between the *An*STRIPAK complex and two MAPK pathways, involved in development and stress responses.

## Results

### The *An*STRIPAK complex is a heptameric protein complex

**Fig 1:**
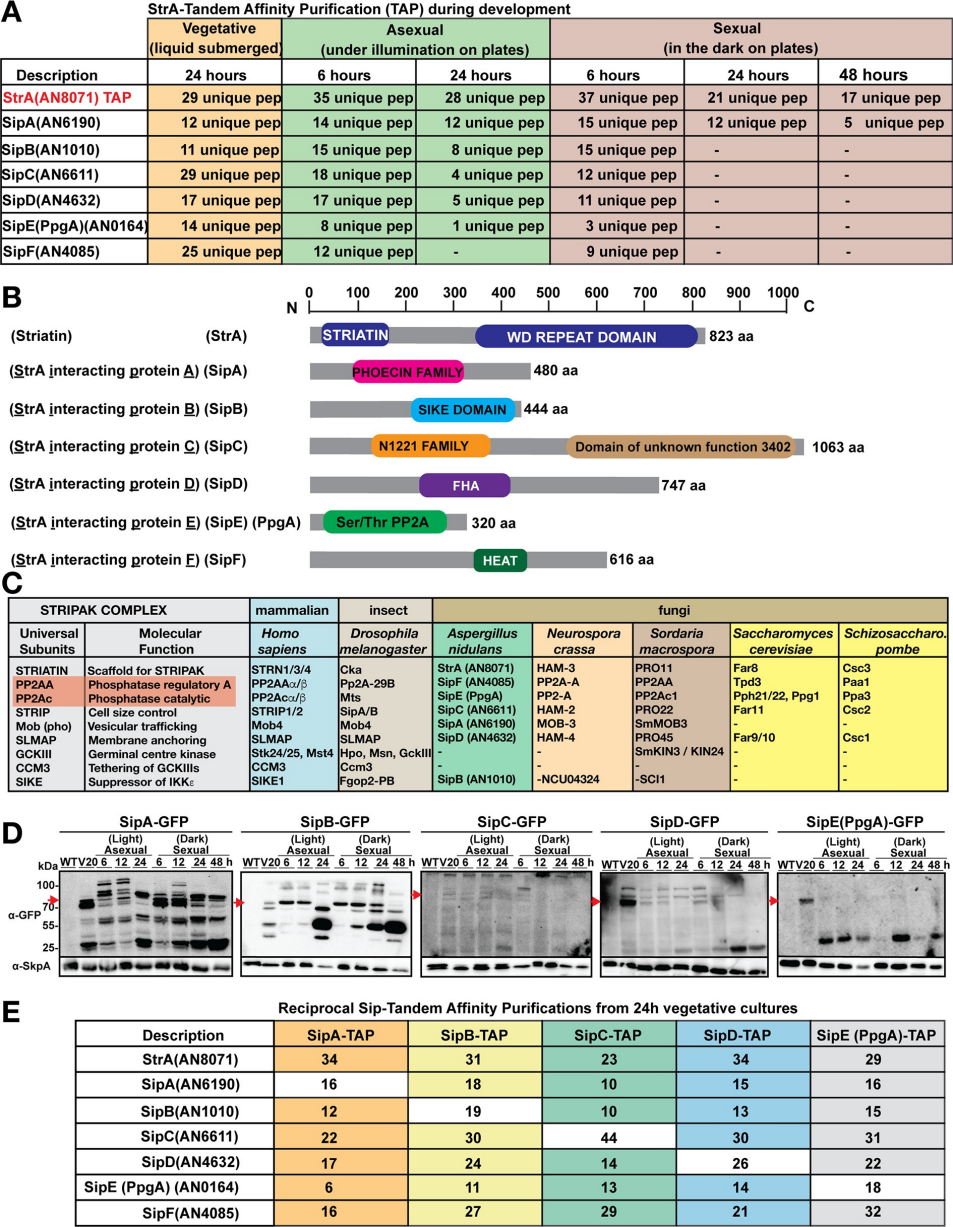
Discovery and composition of the *An*STRIPAK complex. (A) Identification of StrA-interacting proteins (Sips) by tandem affinity purification (TAP). A strain expressing a functional StrA-TAP fusion was grown in liquid GMM (grown vegetatively, i.e. neither sporulation or the sexual cycle are initiated in liquid cultures) (24h). For induction, cultures were grown vegetatively for 20h and shifted on plates to be induced for both light (6 and 24h) and dark (6, 24 and 48h) development by growing them further at 37°C. Therefore, light and dark cultures also represent SM production phase. TAP-MS was performed as described in materials and methods. Unique peptides numbers are given (S1–S6 Tables). (B) Domain structure of the STRIPAK complex members: StrA, SipA, SipB, SipC, SipD, SipE (PpgA) and SipF. N-C; N- and C-terminus, (aa) Number of amino acids. WD (Trp-Asp) repeats, Mob3: Monopolar spindle one-binder protein (Phocein); N1221: acidic domain with possible transmembrane domains, DUF3402; FHA: Forkhead-associated domain, HEAT repeats. (C) Orthologs of STRIPAK complex from yeast to human. The table was mainly adapted from yeast [6] and *A*. *nidulans* STRIPAK orthologs were highlighted. Detailed accession numbers are given in S7 Table. (D) Expression patterns of SipA to SipE (PpgA) proteins during fungal development. Vegetatively grown cells (24h) were shifted onto plates and induced for asexual (6, 12, 24h) and sexual (6, 12, 24, 48h) development. Sip-GFP fusions (100 μg total protein) were used for time course immunoblotting. An α-GFP antibody was used to detect fusions and α-SkpA shows equal loading in each lane. (E) Confirmation of components of the STRIPAK complex by reciprocal TAP-LC-MS/MS. Functional Sip-TAP fusions, grown vegetatively for 24h, were subjected to TAP-LC-MS/MS. TAP tagged proteins are given at the top of the table and proteins copurified are given on the left-hand side of the table. Unique peptides are given as numbers (S8–S12 Tables).

To reveal and understand the molecular interaction network of StrA (AN8071), a fully functional StrA-TAP fusion expressed from its native locus under the control of the endogenous promoter was used for tandem affinity purification (TAP) and liquid chromatography-mass spectrometry (LC-MS/MS) (Fig 1A, S1–S6 Tables). Six proteins associated with StrA, termed striatin interacting proteins (Sips), were identified from vegetative cultures grown for 24 hours: AN6190/SipA, AN1010/SipB, AN6611/SipC, AN4632/SipD, AN0164/SipE (PpgA) and AN4085/SipF. For consistency, SipE and PpgA refer to the same protein within the text. StrA was expressed at higher levels during early stages of asexual and sexual development in comparison to vegetative growth (S1 Fig). Therefore, TAP-MS/MS was performed from light and dark induced cultures (6, 24, 48h). StrA interacted with the same set of proteins SipA-F during early sexual and asexual development (6h). However, it recruited only SipA at late developmental time points (24, 48h) (Fig 1A).

With the exception of SipB, all other interacting partners of StrA are conserved components of the STRIPAK complex in fungi (S7 Table). SipA is an ortholog of human and fungal Mob3 (Phocein) with 480 amino acids (Fig 1B and 1C). Mob3p is a kinase co-activator and a part of the STRIPAK complex in humans, fruit flies and filamentous fungi [15]. Interestingly, The C-terminus of SipB (444 aa) contains a suppressor of IKKε (SIKE)-like domain (S2 Fig), an ortholog of which has only recently been shown to exist in *S*. *macrospora* [17]. SIKE-like proteins contain a coiled-coil domain which is conserved in fungi. Alignment of two human SIKE isoforms with the c-terminus of *A*. *nidulans* SipB indicates 46% similarity between the proteins (S2 Fig).

SipC (1063 aa) is the largest of all with two putative domains, an N1221family domain and a domain of unknown function (DUF3402). It is an ortholog of human STRIP1/2, *Nc*HAM-2 and *Sc*Far11. SipD (747 aa) is an ortholog of human SLMAP, *Nc*HAM-4, *Sm*PRO45 and *Sc*Far10. Both proteins HAM-4 and Far10p control similar developmental events as HAM-2 and Far11p. SipE (PpgA) (320 aa), the smallest of all, is an ortholog of the catalytic subunit of human phosphatase (PP2Ac), *Nc*PP2Ac and *Sc*Pppg1. SipF (616 aa) is the homolog of the human phosphatase regulatory subunit (PP2AA) with fungal homologs *Nc*PP2AA, *Sc*Tpd3, *Sp*Paa1, respectively.

Since SipA interacted with StrA during all developmental stages, expressions of the functional Sip-GFP fusions (S3 Fig) were monitored during vegetative, asexual and sexual stages (Fig 1D). The catalytic subunit SipE (PpgA), but not the regulatory subunit of phosphatase (SipF) was used for expression studies since *sipF* deletion was lethal. A 79kDa SipA-GFP and a 76kDa SipB-GFP fusion were present during almost all developmental time points. A 145kDa SipC-GFP fusion was poorly expressed during developmental stages. A 107kDa SipD-GFP fusion was constantly expressed at all stages except for late sexual development (24 and 48h). Interestingly, the 63kDa SipE-GFP fusion was only present during vegetative growth and degraded and disappeared at both asexual and sexual stages.

In order to define the core STRIPAK complex more precisely, reciprocal TAP-MS/MS was performed. TAP of SipA recruited StrA along with SipB to SipF (Fig 1E). Similarly, TAP of SipB, SipC, SipD or SipE also recruited all members of the complex (S8–S12 Tables). These interactome data clearly underline that a heptameric STRIPAK complex made of a striatin StrA, a Mob3 kinase ortholog SipA, a SIKE-like protein SipB, a STRIP1/2 ortholog SipC, an SLMAP ortholog SipD, and the phosphatase subunits SipE (PpgA) and SipF exists in *A*. *nidulans*.

### The *An*STRIPAK complex is required for proper development and light response

**Fig 2:**
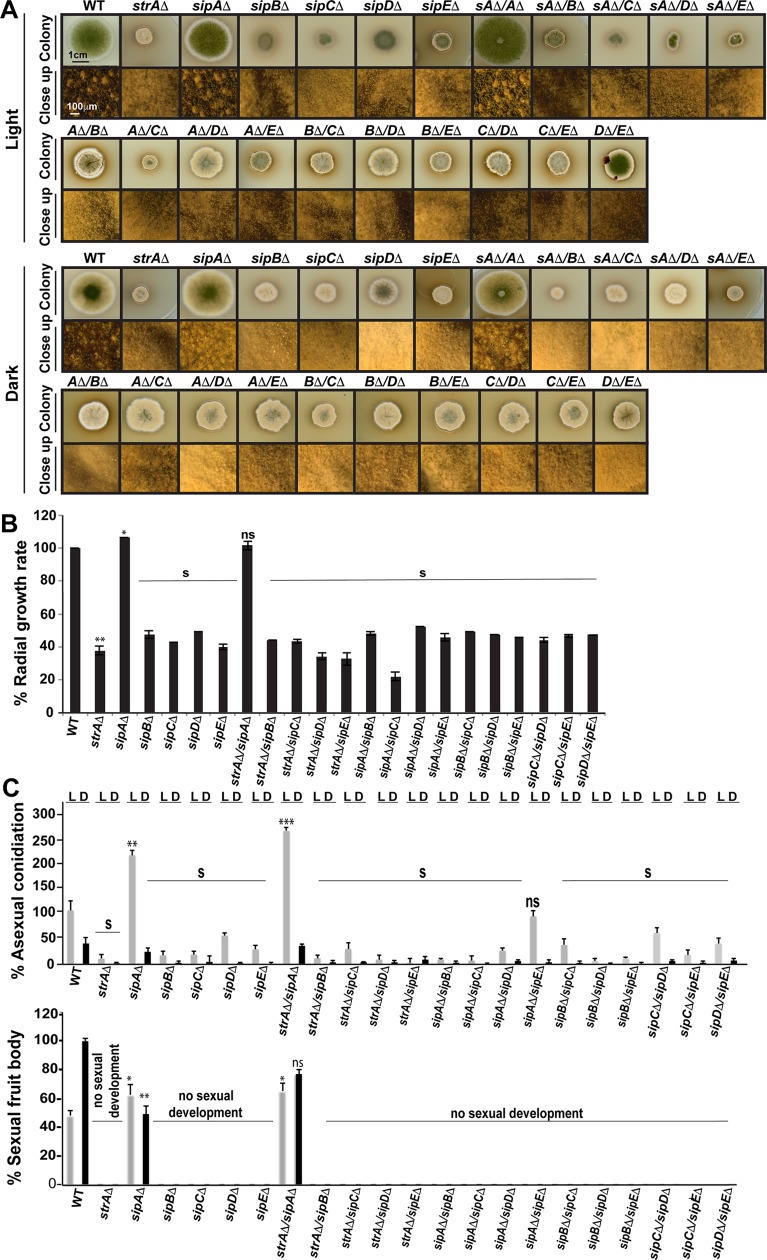
Control of light-dependent fungal development by the *An*STRIPAK complex. (A) Light and dark induced development of single and double mutants of the STRIPAK complex. 5x10^3^ fungal spores were point inoculated on GMM plates and incubated for 5 days at 37°C under constant light (90 μWm^2^) or dark conditions. Upper squares show colony development (scale 1 cm) from plates and lower squares are close-up images of the colonies (scale 100 μm). Whitish round structures represent fruit bodies. *sA*Δ represents *strA*Δ, *A*Δ represents *sipA*Δ, *B*Δ represents *sipB*Δ, *C*Δ; *sipC*Δ, *D*Δ; *sipD*Δ, *E*Δ; *sipE*Δ. For consistency, *ppgA*Δ was presented as *sipE*Δ (B) Quantification of comparative radial growth rate of the single and double mutants from light induced plates. NS; not significant (p > 0.05), S; significant (p < 0.05). WT growth rate serves as 100% standard. (C) Quantification of asexual and sexual development from single and double STRIPAK mutants. L; light, D; Dark, S; significant. Asexual sporulation of WT in light represents 100% sporulation. Sexual fruit body formation of WT in dark was used as 100%. Values are the means of three replicates, and error bars represent standard errors.

To understand the roles of the *An*STRIPAK complex in fungal development, individual *sip* deletions, *sip;sip* double deletions and combinations of *sip* deletions with *strA*Δ were created (S4 Fig) and subjected to developmental tests (Fig 2). Loss of *sipB*, *sipC*, *sipD* and *sipE* all resulted in similar phenotypes to that seen in the *strA*Δ strain, characterized by slower growth rate, reduced conidiation and lack of fruit bodies. *sipE* (*ppgA*), encoding one of the two phosphatase 2A catalytic subunits of *A*. *nidulans* was shown to influence growth together with more than twenty other phosphatases [27]. All attempts to delete *sipF* encoding PP2A-A regulatory subunit failed, suggesting that the gene is essential for viability of *A*. *nidulans* as it is in *N*. *crassa* [15].

**Fig 3:**
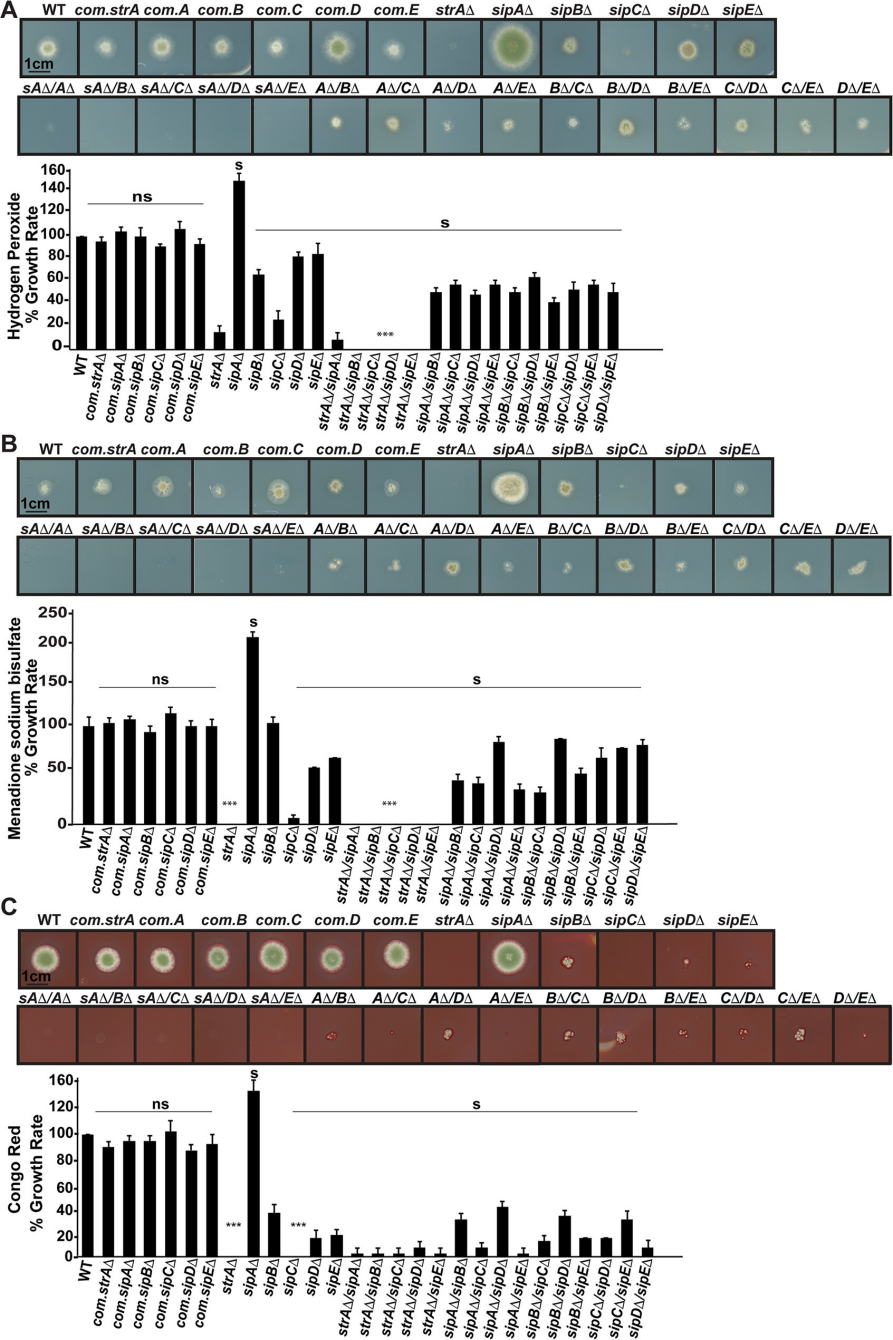
Control of reactive oxygen species (ROS) and cell-wall stress responses by the *An*STRIPAK complex. (A) Sensitivity tests of *An*STRIPAK mutants and mutants complemented with the respective WT alleles (com.*strA*Δ, com.*sipA*Δ, com.*sipB*Δ, com.*sipC*Δ, com.*sipD*Δ, com.*sipE*Δ) in the presence of hydrogen peroxide (H_2_O_2_, 1 mM) measured as radial growth rate. (B) Oxidative stress responses of the strains to Menadione (0.08 mM). (C) Growth of the mutants in the presence of cell-wall stress agent Congo red (20 μg/ml). The cultures (5x10^3^ spores) were grown on solid GMM with stress agents for 5 days at 37°C in light. Scale bar 1 cm. These experiments were repeated at least three times with the same results. Chart graphs show radial growth diameter compared with the WT, which was used as standard (100%). Data are indicated as average ± SD of three independent biological repetitions. Columns with (ns) denote non-significant but (n) denote significant difference and also (***) represent values of strong significant difference p < 0.0001 compared with WT.

The defects were complemented by introducing the corresponding genes into the deletion strains (Fig 3). Furthermore, *sip*Δ*/sip*Δ double deletions and *sip*Δ/*strA*Δ double deletions were similar to single deletion strains. Surprisingly, *sipA*Δ showed an opposite phenotype with significantly increased radial growth and two-fold more conidiation than the WT and production of normally shaped fruit bodies, which were devoid of ascospores (Fig 2). Moreover, *strA*Δ/*sipA*Δ phenocopied *sipA*Δ, suggesting an epistatic effect of *sipA* over the *strA*. In contrast, *sipA* was not epistatic to *sipB* to *sipE* since *sipA* double deletion combinations with other *sip* genes behaved similar to single *sip* deletions, showing that *sipA* is only epistatic to StrA yet not the other members of the *An*STRIPAK complex. These results show that almost all members of the *An*STRIPAK complex are equally important for growth and light-dependent development except for SipA. However, SipF is likely essential for the viability of *A*. *nidulans*.

### The *An*STRIPAK complex is involved in responses to various environmental stress factors

The STRIPAK complex mutants showed developmental defects, suggesting an essential role of the complex in signal transduction of developmental processes. In order to see whether the *An*STRIPAK complex is also involved in stress responses, mutants were subjected to various stress conditions (Fig 3). The radial growth of all single and double deletions except for *sipA*Δ were significantly reduced in oxidative stress (H_2_O_2_, Menadione) and cell wall stress (Congo Red) media in comparison to WT (Fig 3A–3C). *sipA*Δ was more resistant to oxidative and cell wall stressors than the WT. However, under stress conditions *sipA*Δ did not show epistatic effects over the *strA*Δ since the *sipA*/*strA* double mutant was as sensitive as the *strA* single deletion and the double deletions of *strA* and *sip* genes to both stress conditions.

Strains were also monitored to see how they cope with DNA damage, amino acid starvation (3-AT), caffeine and osmotic stress. Similar to oxidative and cell wall stress, *sipA*Δ displayed more robust vegetative growth than the WT under all tested conditions. *strA* double deletions with *sip* genes, interestingly including the *strA*/*sipA* double mutant, were extremely sensitive to all three types of DNA damage conferred by HU, MMS and EMS (S5 Fig). Amino acid starvation mainly influenced *strA/sip* double deletions (S6 Fig). All of the deletion strains excluding the *sipA* deletion were slightly sensitive to osmotic stress induced by NaCl.

These data clearly indicate that the lack of *An*STRIPAK complex results in drastic problems in combating various types of stressors. Furthermore, the epistatic effect of SipA over the StrA deletion is abolished in the presence of stress conditions, suggesting an interplay between SipA and StrA in regulating stress responses.

### The *An*STRIPAK complex controls expression of developmental regulators and reactive oxygen species (ROS) scavenging enzyme genes

**Fig 4:**
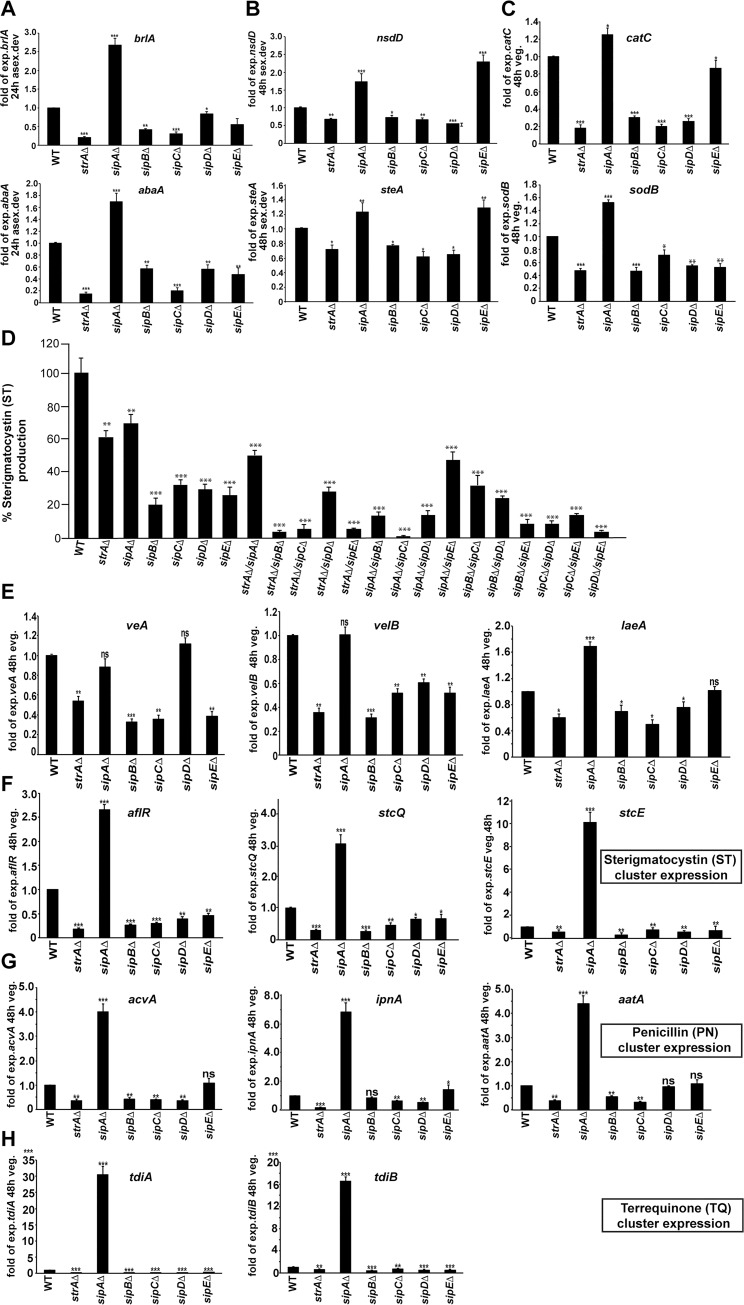
Regulation of developmental and secondary metabolite gene expression by the *An*STRIPAK complex. (A) Transcripts of asexual regulatory transcription factors *abaA* and *brlA* in STRIPAK mutants and the WT after 24h asexual development by qRT-PCR. (B) Expressions of sexual regulatory transcription factors *nsdD* and *steA* in mutants and WT after 48h sexual development. (C) Expressions of catalase, *catC* and superoxide dismutase, *sodB* in STRIPAK mutants after 48h vegetative growth. (D) Production of the mycotoxin Sterigmatocystin (ST) in STRIPAK mutants by HPLC. 5x10^3^ spores were point-inoculated at the center of the GMM plates and incubated in light at 37°C for 5 days. (E) Expression of the velvet complex genes, *veA*, *velB* and *laeA* in STRIPAK mutants. (F) ST gene cluster expression; regulatory *aflR*, two structural genes *stcQ*, *stcE* in the mutants. (G) Penicillin (PN) gene cluster, *acvA*, *ipnA* and *aatA* expression. (H) Terrequinone (TQ) gene cluster, *tdiA* and *tdiB* expression. Experiments were carried out in biological triplicates. The statistical significance is indicated as (*) p < 0.05, (**) p < 0.001, (***) p < 0.0001 and non-significant (ns) p > 0.05 compared with WT.

All mutants of the *An*STRIPAK complex, except for *sipA*Δ, exhibited drastic changes in asexual and sexual development, both of which are regulated by a cascade of transcription factors. We consequently determined the effects of our deletions on expression of these transcription factors by qRT-PCR. Expression of major transcription factors that control conidiophore (*abaA*, *brlA*) and sexual (*nsdD*, *steA*) development were significantly reduced in the STRIPAK mutants except for *sipA*Δ (Fig 4A and 4B) which caused 3-fold higher *brlA*, *abaA* and 2-fold *nsdD*, *steA* expression. This increase was consistent with the increased asexual development of the *sipA* mutant. *An*STRIPAK complex mutants were sensitive to oxidative stress. Therefore, expression of two ROS scavenging enzyme encoding genes, catalase *catC* and superoxide dismutase *sodB* were monitored in the mutants. Expression of both genes was significantly decreased in all *An*STRIPAK mutants except for *sipA*Δ (Fig 4C), where they were slightly upregulated, which was consistent with the higher resistance of this strain to stressors when compared with WT. These expression data demonstrate that the *An*STRIPAK complex is required for the balanced expression of developmental regulators and ROS scavenging enzyme genes.

### The *An*STRIPAK complex plays a key role in regulation of secondary metabolite production

A defect in fungal development particularly in fruit body formation (sexual development) is often associated with changes in SM production. All members of *An*STRIPAK complex are involved in growth and fruit body formation, excluding the SipA protein which is specifically required for ascospore formation in fruit bodies in *A*. *nidulans*. Therefore, levels of the fungal mycotoxin sterigmatocystin (ST) were measured in mutants by HPLC (Fig 4D). Production of ST was significantly reduced in mutants in comparison to the WT. *strA* and *sipA* mutants produced less ST than WT but more than *sipB*, *sipC*, *sipD* and *sipE* (*ppgA*) mutants. ST production of the *strA*/*sipA* double mutant was slightly less than the respective single mutants. However, double deletion of *strA* with *sipB*, *sipC*, or *sipE* resulted in extremely reduced ST (less than 10% of WT).

**Fig 5:**
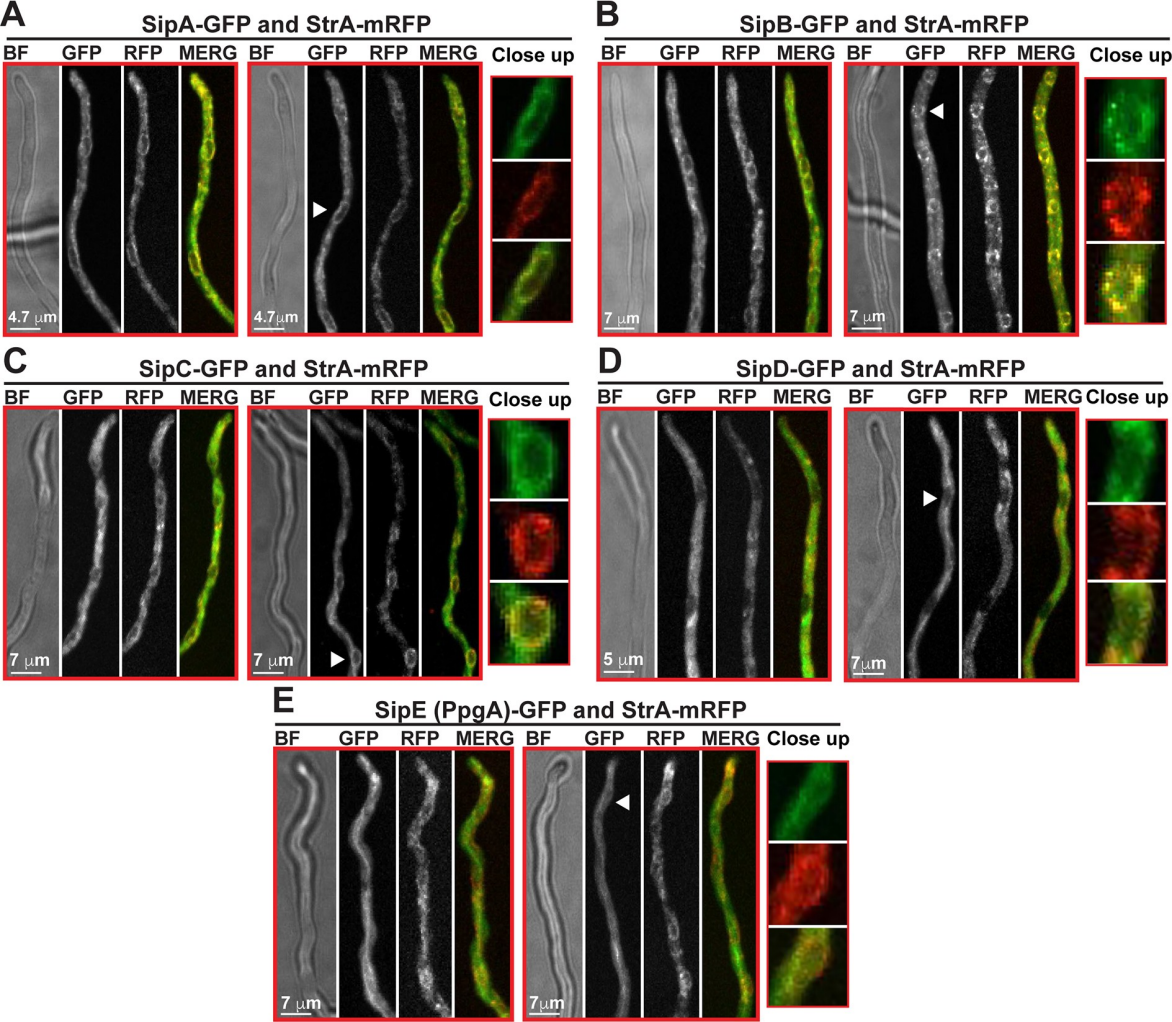
Co-localization of the *An*STRIPAK complex at the nuclear envelope and endoplasmic reticulum. (A) Subcellular co-localization of StrA fused to monomeric red fluorescent protein (mRFP) in combination with SipA-GFP. (B) StrA-mRFP with SipB-GFP. (C) StrA-mRFP with SipC-GFP. (D) StrA-mRFP with SipD-GFP. (E) StrA-mRFP with SipE-GFP. Cells were grown in liquid media at 30°C (i.e. vegetatively) and imaged 17–20 hours after inoculation. Fluorescence images are maximum intensity projections of through focus series. Bright field (BF) images are single focal plane images from the same field. White arrows indicate the position of nuclei around which StrA-mRFP shows ring-like accumulations together with Sip-GFP fusions and the close-up sections. All genes were expressed in their original loci and under their native promoter.

In order to examine if the drastic alterations in SM production in *An*STRIPAK mutants were due to changes in transcript levels of the SM regulators, the expression profiles of the velvet complex along with ST gene cluster as well as two additional SM clusters were determined. Expression of the velvet complex (*veA*/*velB*/*laeA)* was generally diminished in the absence of *An*STRIPAK complex (Fig 4E). The most drastic decrease was observed in *velB* and *veA* expression. The reduced expression of the velvet complex is translated into expression of the ST gene cluster since expression of the transcription factor *aflR* and the two structural genes *stcQ* and *stcE* sharply dropped in *An*STRIPAK mutants except for *sipA*Δ (Fig 4F). Antibiotic penicillin (PN) and antitumour terrequinone (TQ) genes were tested in addition. Surprisingly, expression of *acvA*, *ipnA* and *aatA* required for PN production was reduced in *An*STRIPAK mutants. *ipnA* expression in *sipB*Δ and *aatA* expression in *sipD*Δ did not change (Fig 4G). Similarly, *tdiA* and *tdiB* genes of the TQ cluster were significantly down-regulated in *An*STRIPAK mutants except for the *sipA* mutant, which was higher (Fig 5E). These metabolite and expression data reveal that the *An*STRIPAK complex is important for production of ST and expression of the velvet complex. Furthermore, full expression of at least three different gene clusters ST, PN and TQ require an intact *An*STRIPAK complex, except for *sipA*.

### Localization of the *An*STRIPAK complex on the nuclear envelope is dependent on striatin

**Fig 6:**
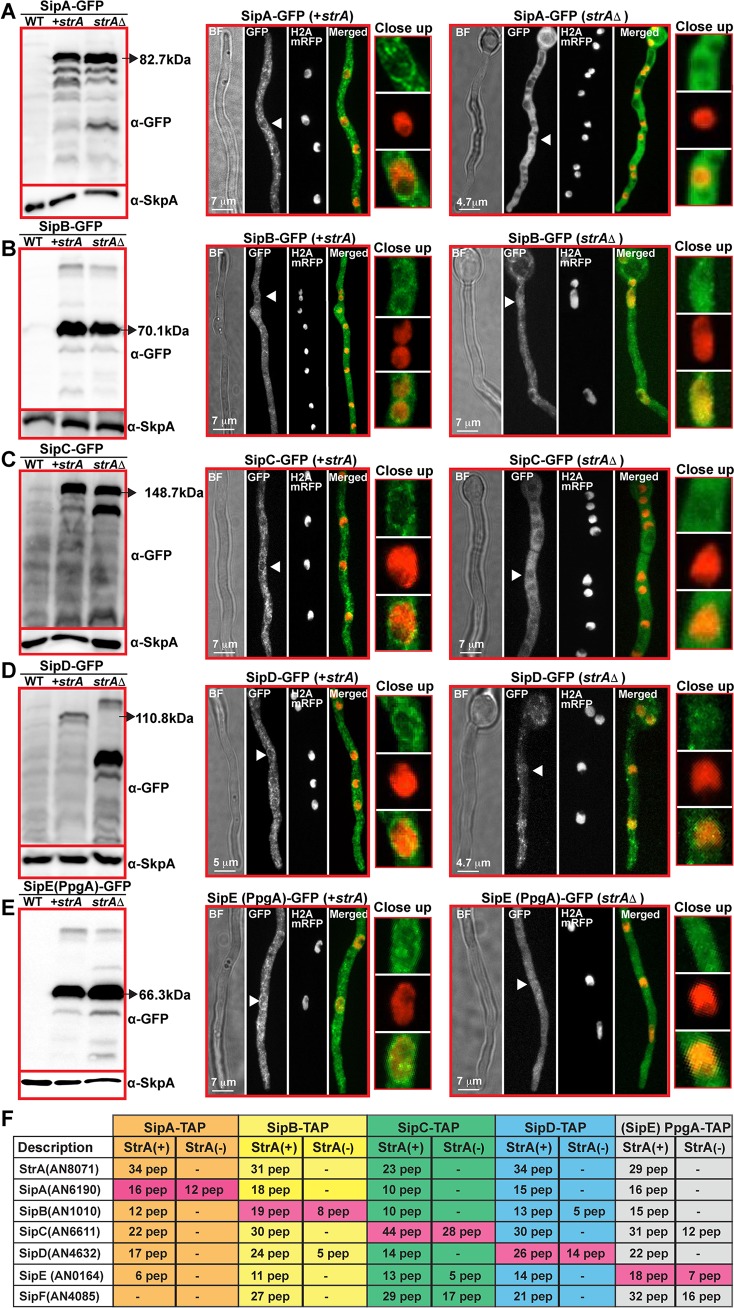
StrA acts as a scaffold for assembly of the *An*STRIPAK complex on the nuclear envelope. (A) Protein levels and subcellular localization of SipA-GFP fusion in the presence (+*strA*) and absence of striatin (*strA*Δ). (B) Protein levels and subcellular localization of SipB-GFP fusion in the presence (+*strA*) and absence of striatin (*strA*Δ). (C) Influence of StrA on SipC, (D) on SipD and (E) SipE expression and localization. (F) Effect of StrA on complex formation of the STRIPAK subunits by TAP-LC-MS/MS. For immunoblotting and microscopy, strains were grown in liquid media for 20h at 30°C and for imaging they were grown at 30°C and imaged 17–20 hours after inoculation. 100 μg crude extract was applied on each lane. GFP fusions and SkpA are detected by α-GFP and α-SkpA. Black arrows show the full length of the Sip-GFP fusion proteins. Fluorescence images are maximum intensity projections of through focus series. Bright field images are single focal plane images from the same field. H2A-mRFP fusions indicate the nuclei. White arrows show the close-up sections from each image. Sip-TAP-LC-MS/MS were performed from 24h vegetative growth in liquid GMM in the presence and absence of StrA. Proteins and number of unique peptides are given in table.

Striatin (StrA) is localized to the nuclear periphery and endomembrane systems in *A*. *nidulans* [26] (S1 Fig). However, it is not known if the entire STRIPAK complex is also associated with the nuclear envelope in *A*. *nidulans*. We have found that SipA to SipF interact with StrA constituting the heptameric *An*STRIPAK complex. A functional StrA-mRFP fusion was co-expressed with SipA, SipB, SipC, SipD and SipE-GFP fusions (Fig 5). The StrA-mRFP fusion, which clearly decorated the nuclear periphery where the nuclear envelope is found, was not primarily found inside the nucleus. Furthermore, StrA was also present on long string-like extensions, presumably representing endomembrane systems such as endoplasmic reticulum. The SipA-GFP fusion was also found to be accumulated around the nucleus and in string-like extensions but was present in the nucleus at trace levels and overlapped with StrA-mRFP signals (Fig 5A). Similarly, GFP fusions of SipB and SipC were found to be co-localised with StrA around the nucleus (Fig 5C). In co-localizations with histone H2A, SipD-GFP and SipE-GFP showed clear perinuclear localization, very similar to those of SipA-GFP, SipB-GFP and SipC-GFP (Fig 6). Colocalizations of SipD and SipE (PpgA)-GFP with StrA-mRFP showed a somewhat different pattern. The staining was less punctate and the localization around the nucleus was visible but weaker than that of SipA- to SipC-GFP (Fig 5D and 5E). We speculate that the mRFP tag on StrA weakens the binding of SipD and SipE to StrA.

Like StrA, SipA to SipE were all co-localized with StrA around the nuclear envelope and partially in endomembrane systems. Since *sip* double deletions with *strA* led to more sensitive phenotypes suggesting the key role of StrA for the molecular function of the *An*STRIPAK complex, SipA to SipE-GFP fusions were expressed in a strain devoid of StrA (Fig 6). The absence of StrA did not influence expression of the Sip proteins except for SipD, which showed a higher molecular weight as well as a thicker lower molecular band (Fig 6D). Interestingly, lack of StrA led to loss of nuclear envelope localization of SipA, which became more diffuse in the cytoplasm. SipB-GFP also lost its nuclear envelope localization in the absence of StrA and dispersed in the cytoplasm and, interestingly, was present in the nucleoplasm except for the nucleolus (Fig 6B). SipC also dispersed from the nuclear envelope and was relatively uniformly distributed in the cytoplasm but it was at least partially excluded from nuclei (Fig 6C). SipD remained punctate in the absence of StrA, but it was no longer concentrated at the nuclear envelope, and many punctae were seen in the nucleoplasm (Fig 6D). SipE (PpgA) showed a similar localization pattern to SipB in the absence of StrA, diffuse in the cytoplasm and nucleoplasm but excluded from the nucleolus (Fig 6E). In summary, our expression data and confocal imaging data reveal that all of the members of the *An*STRIPAK complex localize to the nuclear envelope and endomembrane system. They require the molecular scaffold StrA for normal localization and all but SipD disperse in the absence of StrA. SipD does not disperse in the absence of StrA but its localization pattern is altered.

### Lack of Striatin disrupts the assembly of the *An*STRIPAK complex and reveals subcomplex dynamics

Given the fact that StrA is required for appropriate cellular localization of the *An*STRIPAK complex, it became intriguing to ask whether the mislocalizations reflect the interdependent interactions of the complex proteins. As assayed by TAP purification followed by MS, SipA to SipE all complexed with each other with high peptide numbers in the presence of StrA (Fig 6F, S13–S17 Tables). However, surprisingly, in the absence of StrA, TAP purification of SipA did not pull down any other members of the complex. SipB and SipD only reciprocally copurified with each other. SipC and SipE (PpgA) reciprocally pulled down the regulatory subunit of phosphatase SipF as well as a second regulatory subunit B (PabA; presumably reflecting a distinct, STRIPAK-independent PP2A complex). Surprisingly, in the absence of SipA, StrA was able to establish a form of the *An*STRIPAK complex lacking only SipA (S18 Table). These TAP data comparing the physical interaction dynamics of *An*STRIPAK complex in the presence and absence of StrA clearly display that (I) SipA only interacts with StrA, therefore it is recruited to the *An*STRIPAK complex via StrA and StrA does not need SipA to establish the *An*STRIPAK complex, (II) SipB-SipD form heterodimers and then presumably associate with StrA, (III) SipC-SipE-SipF form a heterotrimeric subcomplex, which is then attached to StrA to form the fully functional *An*STRIPAK complex.

### Striatin controls two unrelated MAPK-pathways: nuclear accumulation of the stress-sensing MpkC MAP kinase and activation of the development-inducing MpkB MAP kinase

**Fig 7:**
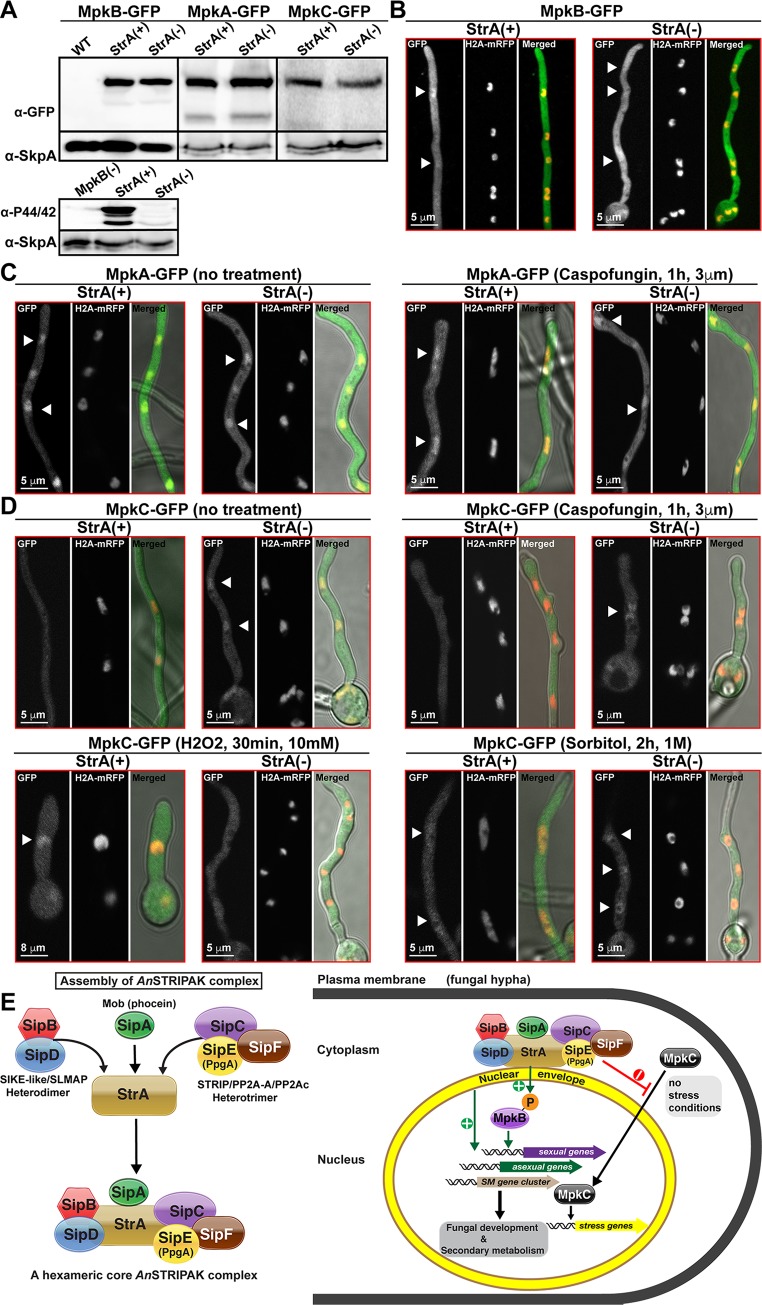
The *An*STRIPAK complex controls nuclear shuttling of the stress MAPK MpkC and activation of MpkB, which is required for development and SM production. (A) Expression of three MAPKs, MpkB, MpkA and MpkC-GFP fusions and activation of MpkB in the presence, StrA (+) and absence, StrA (-) of striatin. α-GFP detects GFP fusions, α-SkpA detects SkpA levels as loading control, α-P44/42 recognizes MpkB phosphorylation. StrA (+) represents WT situation where MpkB is phosphorylated. (B) Cellular localization of MpkB in the presence and absence of StrA. (C) Cellular distribution of MpkA under normal growth (16h veg.) and with the cell-wall damage inducing drug (caspofungin 3 μm, 1h postinduction) treated conditions in StrA (+) and StrA (-). (D) Subcellular localization of MpkC under normal, cell-wall damage induced (caspofungin), oxidative (H_2_O_2_, 10 mM, 30min) and osmotic stress (Sorbitol, 1 M, 2h) conditions. White arrows show the nuclei. (E) Our model of *An*STRIPAK complex assembly and its role in development and stress responses. *An*STRIPAK is made from three subcomplexes: SipB (SIKE-like)-SipD (SLMAP) heterodimer, SipA(Mob3)-StrA(STRN) heterodimer and the SipC-SipE-SipF (STRIP-PP2AA-PP2Ac) heterotrimer, which assemble on the StrA scaffold on the nuclear envelope. StrA prevents nuclear shuttling of stress MAPK MpkC under no-stress conditions to help it to receive proper stress signals in cytoplasm. StrA also promotes phosphorylation of pheromone response module kinase MpkB, which is required for sexual fruit body formation and SM production. *An*STRIPAK complex assembly and regulation controls asexual and sexual development, SM production and stress responses.

It was shown that activation of the *N*. *crassa* cell-wall stress pathway MAPK MAK-1 as well as its nuclear accumulation was reduced in core components of STRIPAK complex mutants [15]. The same study also showed that MAK-2, which regulate hyphal fusions in *N*. *crassa*, phosphorylates MOB-3 component of STRIPAK. Striatin mutants in *A*. *nidulans* show developmental and SM defects. Furthermore, the mutants also show sensitivity to cell-wall and oxidative stressors. Therefore, we wondered how common MAPK pathways are influenced by the STRIPAK complex and whether the influence of STRIPAK on MAPK pathways was similar to that of *N*. *crassa*. In addition to MpkB which controls sexual development and SM production, there are three mitogen activated protein kinases (MAPK): MpkA is mainly responsible for cell-wall regulation, MpkC and SakA (yeast Hog1 ortholog) play roles in stress responses, particularly oxidative and osmotic stress responses [24, 28]. To determine if StrA influences the localizations of these kinases, the three MAPKs, MpkA (cell-wall stress), MpkB (sexual development and SM production) and MpkC (oxidative and osmotic stress) were expressed as GFP fusions in the presence and absence of StrA (Fig 7). All kinases were expressed similarly in the presence or absence of StrA (Fig 7A). Surprisingly, activation phosphorylation (P-44/42) of MpkB, which is necessary for fruit body formation, was almost totally lost in the absence of StrA. MpkB localization, which was not influenced by lack of StrA, was mainly nucleo-cytoplasmic (Fig 7B). Furthermore, MpkB (yeast Fus3p) interacted with MAP2K MkkB (yeast Ste7p), recently characterized scaffold protein HamE and transcription factor SteA (yeast Ste12p) in the absence of StrA (S19 and S20 Tables). In contrast to *N*. *crassa* MAK-1, MpkA exhibited nuclear localization both in WT and *strA*Δ strains, which was not influenced by cell-wall damaging antifungal drug caspofungin (Fig 7C). Interestingly, MpkC, which was mainly found in the cytoplasm under non-stress conditions in the WT, imported into the nucleus in the absence of StrA (Fig 7D). Caspofungin treatment had no influence on the localization of MpkC. Both oxidative and osmotic stress conditions led to nuclear accumulation of MpkC in WT. However, lack of StrA resulted in loss of MpkC nuclear enrichment under oxidative stress whereas osmotic stress had no effect on MpkC localization in the *strA* deletion. These results imply that StrA acts differently in *A*. *nidulans* and mainly prevents MpkC from entering into the nucleus under non-stress conditions to respond to stress and is required for nuclear accumulation of MpkC under oxidative stress conditions. Furthermore, StrA controls phosphorylation of MpkB, which is required for coordination of development and SM production.

## Discussion

The STRIPAK complex is highly conserved in eukaryotes and is involved in many cellular functions [1, 6]. In this study, we have revealed the molecular nature and functions of the *An*STRIPAK complex, which consist of at least seven proteins StrA/STRN, SipA/Phocein, SipB/SIKE1-like, SipC/STRIP, SipD/SLMAP, SipE(PpgA)/PP2Ac and SipF/PP2AA. Detailed phenotypic, genetic, biochemical, live cell imaging and chemical approaches identified StrA as the core scaffolding protein which assembles at least six other members at the nuclear envelope to control intracellular signaling events (Fig 7F). StrA is found in a heterodimer state with SipA/Phocein and further recruits the heterodimer SipB/SipD (SIKE/SLMAP) and the heterotrimer SipC/SipE/SipF (STRIP/PP2Ac/PP2AA) to establish a heptameric complex. This complex controls gene expression for the sexual reproductive cycle, including formation of multicellular fruit bodies and furthermore, it is also a key regulator for production of SMs.

Although the molecular composition of the STRIPAK complex is conserved, the described functions of the complex show diverse roles in fungi, flies and mammals. In the baker´s yeast *S*. *cerevisiae*, the FAR (STRIPAK) complex acts as an antagonist of target of rapamycin (TOR) pathway and counteracts recovery from pheromone arrest [11, 12] whereas in the fission yeast *S*. *pombe*, the STRIPAK complex acts as a negative regulator of septation initiation [13]. In closely related filamentous fungi *S*. *macrospora* and *N*. *crassa*, STRIPAK regulates hyphal fusion and fruit body formation [14, 15]. In two *Fusarium* species, which are plant pathogenic fungi, striatin is required for pathogenicity on host organisms [29, 30]. In the fruit fly, the STRIPAK complex controls epithelial cell movement and tissue size by modulating two different signaling pathways [31, 32], whereas in the nematode *C*. *elegans*, members of the complex control polarity establishment during embryogenesis [33]. In humans, STRIPAK complex governs embryonic stem cell differentiation, proper cardiac function, dendritic spine morphology and cancer [34, 35].

Lack of fully assembled *An*STRIPAK complex results in loss of proper light response as a result of defective asexual and sexual development. The *An*STRIPAK complex controls light-dependent fungal development. In this fungus, light controls asexual reproduction through the various light receptors. The velvet complex physically and functionally interacts with the red and blue light receptors [36]. Improper expression of the velvet complex in STRIPAK mutants might influence interaction dynamics of the complex with light receptors, therefore, disrupting the light-dependent development. Particularly, two major asexual transcription factors *abaA* and *brlA* are induced by *An*STRIPAK complex, which drives asexual responses. The role of *An*STRIPAK complex in sexual development might be somewhat complicated. It controls formation of fruit bodies by properly dosing expression of sexual transcription factors such as *nsdD* and *steA*. It is known from *N*. *crassa* and *S*. *macrospora* that the STRIPAK complex is involved in cellular fusion, which finally leads to fruit body formation in these fungi. In *A*. *nidulans*, fruit body formation is also controlled by formation of cell-cell fusions. Loss of fruit bodies also indicate that there are defects in cell-cell fusions in the absence of *An*STRIPAK. In *N*. *crassa*, the NRC-1-MEK-2-MAK-2 kinase cascade are the central components of self signalling machinery [37]. The MOB-3 component of the *Nc*STRIPAK complex interacts with MAK-2. However, nuclear localization of MAK-2 is not influenced by STRIPAK but MAK-1 localization is altered. In *A*. *nidulans*, the sexual pathway is controlled by a pheromone response (SteC-SteD-MkkB-MpkB) module which migrates from the plasma membrane to the nuclear envelope to deliver MpkB into the nucleus. Phenotypes of MAPK module mutants are very similar to STRIPAK mutants [24]. Interestingly, in time course purifications at sexual stages, SteD, which is the adaptor domain of the pheromone response pathway, was repeatedly co-purified with the StrA-TAP fusion (S1–S6 Tables). Both MkkB and MpkB localizations did not change and MpkB interacted with both MkkB and SteA in the absence of StrA. However, interestingly, MpkB was not activated by phosphorylation in the absence of StrA. Since MpkB interacts with SteA-VeA and phosphorylates VeA, which is necessary for sexual development and SM production, the *An*STRIPAK complex presumably activates fruit body formation and SM production by phosphoactivation of the MAPK module.

The *sipA* mutant and its *strA* combinations showed an opposite phenotype to STRIPAK component deletions, indicating an epistatic function of SipA over the StrA but not over the SipB-SipC-SipD-SipE complex. The *sipA* deletant grows as well as WT or even better. It produces 2.5-fold more asexual spores and is more resistant to different stress conditions. SM production was slightly influenced in the absence of *sipA* and expression of all other gene clusters was upregulated in *sipA*Δ, which strongly suggests an inhibitor role for SipA in these processes. How does SipA perform this function within the *An*STRIPAK complex? This is obvious from interaction dynamics, because SipA interacts only with StrA during all developmental stages. Furthermore, in the absence of SipA, StrA is able to form a hexameric complex with SipB, SipC, SipD, SipE and SipF. This partial *An*STRIPAK(-SipA) complex without SipA is presumably more active than an intact complex, and it promotes excessive growth and asexual sporulation by unknown mechanisms. However, it does not sufficiently fulfill the meiotic functions of the WT complex, because the *sipA* deletant cannot produce ascospores. MOB-3 (SipA homolog) in *N*. *crassa* interacts with the NRC-1-MEK-2-MAK-2 kinase self-signaling cascade [38]. However, although SipA does not interact with the components of the MAPK pathway in the absence of StrA in TAP studies, it might transiently interact with the MAPK pathway to elicit its effect on development. The partial complex functions (*An*STRIPAK(-SipA)) will require more understanding at experimental level.

In *N*. *crassa* and *S*. *macrospora*, the involvement of the STRIPAK complex in SM has not been reported. Production of the mycotoxin ST is positively controlled by the *An*STRIPAK complex, which requires proper expression of the velvet complex. Accordingly, the expression of the ST gene cluster is also drastically reduced by loss of the *An*STRIPAK complex. Expression of PN and TQ clusters are similarly diminished in the STRIPAK mutants. *A*. *nidulans* produces many more metabolites than these three molecules. Although only three gene clusters were examined here, the effects of the loss of STRIPAK might be more extensive. How does the complex control SM production? Transcriptional downregulation of the velvet complex might be the primary reason why SM production is drastically affected in STRIPAK mutants. Another scenario might be that *An*STRIPAK is important for vital functions of the SteC-SteD-MkkB-MpkB module. Because this module uses the nuclear envelope to interact with the nucleus and deliver the active MAPK MpkB into the nucleus. As discussed previously, reduction in signal fidelity of the pheromone response pathway in the absence of StrA presumably results in reduced SM production.

In other eukaryotes, STRIPAK complex acts as a negative regulator of kinases, because the GCKIII kinase family member Mst3 and Mst4 are hyperphosphorylated in the mutants of PP2A subunit in human cells or okadaic acid treated cells, respectively [39]. The scenario in *A*. *nidulans* and *N*. *crassa* is different, however. In *A*. *nidulans* MpkB loses its phosphorylation in the absence of striatin and MpkB is a MAPK, not a GCK type kinase. In contrast, in *N*. *crassa*, MAK-1 loses its activity under resting and stress conditions [15]. Two GCKs SmKIN3 and SmKIN24 were found to be functionally and physically interacting with *S*. *macrospora* striatin [40, 41]. However, functional control of these kinases by striatin remains to be shown.

The SIKE-like domain containing SipB is part of the *An*STRIPAK complex and it forms a heterodimer with SipD (SLMAP), which is similar to mammals where SIKE and SLMAP form a heterodimer. However, it is not surprising to see a mammalian anti-inflammatory protein conserved in fungi, including *A*. *nidulans* since it was also shown that velvet family proteins of *A*. *nidulans* contain a DNA binding domain structurally similar to the proinflammatory Rel family of NF-KB proteins although they show only 13% similarity at the amino acid level [42]. Presence of NF-KB like velvet family proteins as well as SIKE-like proteins in fungi strengthens the hypothesis that fungal and mammalian defense systems have some degree of analogy since SMs of fungi often act as a defense mechanism against competitors or predators [43]. A SIKE-like protein as part of the *An*STRIPAK complex participates in production of SMs such as the mycotoxin ST to help the fungus to compete with other organisms in soil.

The *An*STRIPAK complex mutants are sensitive to oxidative and cell-wall stress, however they are not extremely influenced by osmotic stress. At least two genes *catC* and *sodB* are down-regulated in the absence of the *An*STRIPAK complex. In *N*. *crassa*, transport of MAK-1 (cell wall regulator kinase) into the nucleus is facilitated by *Nc*STRIPAK complex in a MAK-2-dependent manner. However, the scenario is somewhat different in *A*. *nidulans*. *A*. *nidulans* stress responses are mediated by three MAPK proteins, MpkA, SakA and MpkC. MpkA regulates cell-wall integrity [28]. SakA and MpkC MAPKs control oxidative stress responses interdependently in *A*. *nidulans* [44, 45]. Striatin has no influence on MpkA, which is a homologue of *N*. *crassa* MAK-1. MpkC nuclear accumulation is restricted by StrA in the absence of stress conditions. It is known that MpkC orthologs are activated in the cytoplasm in response to stress and enter into the nucleus. The function of the *An*STRIPAK complex is presumably to keep MpkC in the cytoplasm in the absence of stress, which allows subsequent activation under stress conditions.

In conclusion, this study revealed the composition and assembly hierarchy of the *An*STRIPAK complex. It was surprising that STRIPAK also regulates light and stress responses and the production of SMs. Since *A*. *nidulans* is representative of more than three hundred biotechnologically and medically relevant members of the genus *Aspergillus*, the information gained through this study is applicable to other important fungi to understand their biology and to use their full potential in SM and enzyme production. Paradoxically, deletion of *sipA* resulted in an increase in transcription of a number of SM genes and this deletion may be a useful tool in eliciting expression of silent gene clusters, a goal of genome mining for useful SMs. A functional link between STRIPAK and MAPK pathways has been established and further information on this connection will also reveal how fungi sense various signals and how they control signal influx on the nuclear envelope and how they convert these signals into appropriate responses to control their growth, development and pathogenicity.

## Materials and methods

### Strains, media and growth conditions

Fungal strains used in this study are listed in S21 Table. *A*. *nidulans* AGB551, which has a WT *veA* allele, was used for all deletion and epitope taggings. Stellar (Clontech) and MACH-1 (Invitrogen) competent *Escherichia coli* cells were used for recombinant DNA preparations. The WT and transformed *A*. *nidulans* strains were grown in Glucose Minimal Medium (GMM), supplemented with appropriate amounts of vitamins. For vegetative stage experiments, fungal spores were grown submerged in liquid GMM with 180 rpm rotation for 20 or 48h. For induction of development, vegetative mycelia were then filtered through miracloth and placed on solid GMM with 2% Agar. To induce the cultures asexually, cultures were grown vegetatively for 20h and shifted to the plates and were further incubated in the presence of light for 6, 12, 24h. For sexual induction, plates were covered by aluminium foil and incubated in the dark for 6, 12, 24, 48h. *E*. *coli* were grown in LB broth agar or liquid LB with Ampicillin (100 μg/ml) at 37°C overnight.

### Nucleic acid and plasmid constructions methods

Transformation of *E*. *coli* and *A*. *nidulans* were performed as explained in detail [46, 47]. The plasmids and oligonucleotide sequences used and created in this study are listed in S22 and S23 Tables, respectively.

### Generation of *strA* deletion cassette, complementation and tagging protein-encoding gene with *sgfp*, *mrfp* and *ctap*

In order to create the *strA* deletion construct, the 5’ UTR region of *strA* was amplified from wild-type genomic DNA (AGB551) using primers OZG1025/1110 and 3’ UTR with OZG1112/1028 for *ptrA*. 5’ UTR and 3 UTR were amplified with (OZG1025/1111, OZG1113/1028) for *AfpyroA*. The two fragments were fused to the *ptrA* and *AfpyroA* markers and amplified by a fusion PCR, using oligos OZG1025/1028 (4–4.2 kbp). Both deletions cassettes were cloned in *Sma*I site of pUC19 leading to the plasmids pOB525 (*strA*Δ::*ptrA*) and pOB526 (*strA*Δ::*AfpyroA*). Both plasmids were digested with *Pme*I (*Mss*I) and linear deletion fragments were transformed into AGB551 generating *strA* deletions STRA-DEL1 and STRA-DEL2, respectively. pOB525 was transformed into ANNE21, ANNE22, ANNE23, ANNE24 and ANNE25, resulting in ANNE31, ANNE32, ANNE33, ANNE34 and ANE35 strains.

For complementation of the *strA* deletion, the *strA* genomic locus (6.6 kbp), containing 2 kbp promoter and terminator regions, were amplified from gDNA (NE94/NE95) and cloned into the *Pme*I site of pOSB114, which yielded pNE33. Then, pNE33 was introduced into *strA* deletion strains (STRA-DEL1), generating the ANNE46 strain.

For the creation of *strA*::*sgfp* and *strA*::*ctap* fusions, the promoter and ORF (4.4 kbp) (OZG1025/1026), and terminator sequences (OZG1025/1028) of *strA* were amplified. These two fragments were fused to *sgfp*::*natR*, *ctap*::*natR* cassettes in *Sma*I site of pUC19 by using in-fusion HD cloning kit leading to plasmids pOB480 (*strA*::*sgfp*) and pOB481 (*strA*::*ctap*). In order to create the *strA*::*mrfp* fusion, the promoter as well as ORF of *strA* (OZG1025/1114) and terminator sequences (OZG1115/1028) were amplified and fused to *mrfp*::*AfpyrG* by cloning in *Sma*I site of pUC19 leading to pOB527. These cassettes were released by *Pme*I digestion and introduced into AGB551 strain and ANNE26, ANNE27, ANNE28, ANNE29 and ANNE30. All deletions and epitope tags were confirmed by the Southern blots (S3 and S4 Figs).

### Generation of striatin interacting protein encoding gene, *sipA*, *sipB*, *sipC*, *sipD* and *sipE* (*ppgA*) deletion, complementation, *sgfp* and *ctap* tagging plasmids

For the creation of the *sipA* deletion plasmid construct, the 5’ UTR region of *sipA* was amplified from AGB551 (WT) genomic DNA using primers (NE1/3 and NE1/7) resulting in approximately 1.5 kbp fragments. The 3’ UTR region was amplified with (NE4/N5). The two fragments were fused to the *AfpyrG* and *AfpyroA* markers and inserted into *Sma*I site of pUC19 leading to the plasmids pNE1 (*sipA*Δ::*AfpyrG*) and pNE2 (*sipA*Δ::*AfpyroA*) which were then transformed into MACH-1 competent *Escherichia coli* cells as described previously. Plasmid constructs were isolated using Qiagen Mini-Prep Plasmid Purification (Cat no: 27104) kit. To amplify the deletion cassette, NE2/6 primers were used and 4.2kbp linear fragment was extracted from the gel. These linear deletion fragments were used to transform AGB551, generating the *sipA* deletion strains, termed (ANNE1.1 and ANNE1.2), and StrA-DEL1 for double deletion combinations with *strA* (ANNE1.3). The *sipA* complementation plasmid was generated by amplifying *sipA* genomic locus including, 1.9 kbp promoter and 1.9 kbp terminator regions using primers (NE84/85) and cloned into the *Pme*I site of pOB114 that yielded pNE28, which was introduced into *sipA* deletion strain (ANNE1.1), generating the ANNE41 complementation strain. All deletion strains were verified by Southern hybridization (S4 Fig).

For the creation of *sipA*::*sgfp* and *sipA*::*ctap* fusions, the promoter (NE1/61) and ORF (5.5kbp), along with the terminator sequences (NE5/62) of *sipA* were amplified from gDNA. These two fragments were fused to *sgfp*::*AfpyrG*, *sgfp*::*AfpyroA* and *ctap*::*AfpyrG* cassettes in *Sma*I site of pUC19 by in-fusion HD cloning kit creating pNE13 (*sipA*::*sgfp*::*AfpyrG*), pNE23 (*sipA*::*sgfp*::*AfpyroA*), pNE14 (*sipA*::*ctap*::*AfpyrG*) plasmids respectively. The nest oligos (NE2/6) amplified these cassettes from pNE13, pNE23, and pNE14, which were introduced into the wild-type (ABG551) yielding strains ANNE16, ANNE26, ANNE21. These plasmids were also introduced into *strA* deletion strain STRA-DEL1 as explained in S21 Table.

The construction of *sipB* deletion strains (ANNE2.1 and NANE2.2) was performed as explained above, using the primers NE8/10 and NE8/14 for amplifying the 5’ UTR and NE11/12 for amplifying the 3’ UTR. These fragments were fused to *AfpyrG* and *AfpyroA* markers and inserted into *Sma*I site of pUC19 leading to pNE3 (*sipB*Δ::*AfpyrG*) and pNE4 (*sipB*Δ::*AfpyroA*). The nest oligos NE9/13 were used to amplify deletion cassettes (4.2 kbp), which were ultimately transformed into ABG551 to generate *sipA* deletion (ANNE2.1 and ANNE2.2) and double deletion combinations with *strA* (ANNE2.3), *sipA* (ANNE6), *sipC* (ANNE10), *sipD* (ANNE11), and *sipE* (ANNE12).

The *sipB* genomic locus (5.4kbp), containing the 2kb promoter region and 2 kbp terminator regions, was amplified from genomic DNA (NE86/87) and used for the complementation of *sipB* deletion strains. This fragment was cloned into the *Pme*I site of pOSB114 that yielded pNE29 that was introduced into *sipB* deletion strains to generate ANNE42 strain. The *sipB*::*sgfp* and *sipB*::*ctap* cassettes were generated by using *AfpyrG* or *pyroA* markers in the same way that was explained previously, using primers NE8/63 and NE12/64 and pNE15 /pNE16. The resulting strains were named ANNE17 (*sipB*::*sgfp*::*AfpyrG*), ANNE27(*sipB*::*sgfp*::*AfpyroA*) and ANNE22 (*sipB*::*ctap*::*AfpyrG*).

In order to create ANNE3.1 and ANNE3.2, the 5’ UTR region of *sipC* was amplified from WT gDNA using primers NE15/17 or NE15/21. The 3’ UTR regions were amplified with NE18/19. The two fragments were fused to the *AfpyrG* and *pyroA* markers and inserted into *Sma*I site of pUC19 leading to pNE5 (*sipC*Δ::*AfpyrG*) and pNE6 (*sipC*Δ::*AfpyroA*). Linear deletion cassettes (4.2kbp), which were amplified by PCR, using nest oligos (NE16/20), were transformed to AGB551 generating *sipC* single deletion (ANNE3.1 and ANNE3.2) and double mutant combinations of *sipC* with *strA* (ANNE3.3), *sipA* (ANNE37), *sipD* (ANNE13) and *sipE* (ANNE14). For construction of complementation plasmid, pNE30, a 7kbp *sipC* genomic fragment (NE88/89) was cloned into the *Pme*I site of pOSB114. The pNE30 was then transformed into *sipC* deletion ANNE3.1 Yielding ANNE43 as described previously.

To create pNE17, pNE18 and pNE25, the *sipC* promoter and ORF was amplified with NE15/65 and the terminator region with NE19/66, using AGB551 genomic DNA as a template. These fragments were fused to *sgfp*::*AfpyroA*, *sgfp*::*AfpyrG* or *ctap*:: *AfpyrG* cassettes and cloned in pUC19 as described for the *sipA* and *sipB*. The primers NE16/20 amplified 10 kbp linear fragments from (pNE17, pNE18 and pNE25). These fragments were transformed into the wild type to generate ANNE18, NANE23, NANE28, respectively. Gene replacement events were verified by the Southern hybridization.

For the creation of the *sipD* deletion construct, the 5’ UTR (NE41/42 or NE59/42) and the 3’ UTR (NE43/44) were amplified and fused to the *AfpyrG* and *AfpyroA* markers and inserted into *Sma*I site of pUC19 leading to the plasmids pNE9 (*sipD*Δ::*AfpyrG*) and pNE10 (*sipD*Δ::*AfpyroA*), respectively. Deletion cassettes were amplified with NE45/46 and used to transform AGB551, generating the *sipD* single deletion ANNE4.1 and ANNE4.2 and double deletion combinations with *strA/sipD* (ANNE4.3), *sipA/sipD* (ANNE8), and *sipE*/*sipD* (ANNE15). For complementation of the *sipD* deletion, *sipD* genomic DNA including 1.97 kbp of the promoter region and 2 kbp of the terminator, was amplified (NE90/91) and cloned into pOSB114 at the *Pme*I site, generating pNE31, which was then transformed into ANNE4.1. This yielded the ANNE44 strain.

To generate the *sipD*::*sgfp* and *sipD*::*ctap* strains, the primers NE41/67 were used to amplify the *sipD* promoter and ORF for *sgfp* and *ctap* cassettes, while the primers NE44/68 were used to replicate the *sipD* terminator. These fragments were fused to *sgfp*::*AfpyrG*, *sgfp*::*AfpyroA* and *ctap*::*AfpyrG* with NE45/NE46. Created endogenous fragments (pNE19, pNE26, pNE20) were transformed into AGB551, yielding ANNE19, ANNE29 and ANNE24 respectively.

To generate the *sipE* (*ppgA*) deletion construct, *sipE* 5’ UTR (NE50/51 and NE50/60) and the 3’ UTR (NE52/53) were amplified. The two fragments were fused to the *AfpyrG* and *AfpyroA* markers and inserted into *Sma*I site of pUC19 yielding pNE11 (*sipE*Δ::*AfpyrG*) and pNE12 (*sipE*Δ::*AfpyroA*). The liner deletion fragments, which were amplified with nest oligos NE54/55, then used to transform AGB551, generating *sipE* deletion strains (ANNE5.1 and ANNE5.2, respectively) and double deletions *sipE*/*strA* (ANNE5.3), *sipE*/*sipA* (ANNE9).

For complementation of the *sipE* deletion, the *sipE* genomic locus (6.8 kbp), containing 2 kbp of the promoter region and 2 kbp of the terminator regions, was amplified (NE92/93) cloned into the pOSB114 (pNE32). Then pNE32 was introduced into *sipE* deletion strain (ANNE5.1), generating the ANNE45 strain. To generate the *sipE*::*sgfp* and *sipE*::*ctap* strains, primers NE50/69 were used to amplify the *sipE* promoter and ORF for *sgfp* and *ctap*, while the primers NE71/70 were used to amplify the *sipE* terminator. These fragments were fused to *sgfp*::*AfpyrG*, *sgfp*::*AfpyroA* and *ctap*::*AfpyrG* cassettes with NE54/55. Endogenous fragments (pNE21, pNE27, pNE22) were transformed into AGB551, yielding ANNE20, ANNE30 and ANNE25, respectively.

### Generation of histone 2A mRFP fusion strains

The pOB340 plasmid was digested with *Pme*I at 37°C overnight, then transformed into ANNE16, ANNE17, ANNE18, ANNE19 and ANNE20, resulting in generation of ANNE47, ANNE48, ANNE49, ANNE50 and ANNE51. The same strains were transformed with pME3857, which led to strains ANNE57, ANNE58, ANNE59, ANNE60 and ANNE61. These strains were introduced with pOB526 (*strA*Δ::*AfpyroA*) in order to delete *strA* gene generating the strains ANNE62, ANNE63, ANNE64, ANNE65 and ANNE66.

### Generation of MpkA, MpkB and MpkC::sGFP fusions in *strA* deletion

In order to create MpkB/*strA*Δ combination, pOB526 strA deletion cassette was introduced into AGB655 strain, which yielded ANNE73. Generation of MpkA and MpkC fusions were performed as follows: *mpkA* and *mpkC* promoter and ORFs were amplified (BK546/547 for *mpkA*, BK552/553 for *mpkC*) from gDNA. 3 UTRs for both genes were amplified (BK548/549 for *mpkA*, BK554/555 for *mpkC*) and fused to *sgfp*::*AfpyrG* cassette in *Sma*I site of pUC19, which yielded pBK125 (*mpkA*::*sgfp*::*AfpyrG*) and pBK126 (*mpkC*::*sgfp*:*AfpyrG*), respectively. Both plasmids were transformed into AGB551, which led to ANNE67 (MpkA::sGFP) and ANNE68 (MpkC::sGFP). Then, mRFP::H2A plasmid pME3857 was introduced to ANNE67 and 68, which resulted in ANBK116.1 and 117.2, respectively. Finally, *strA* gene was deleted with pOB526 in ANBK116.1 and 117.2 yielding ANBK119 (MpkA::sGFP/*strA*Δ) and 120 (MpkA::sGFP/*strA*Δ), respectively.

### Hybridization techniques

Southern hybridization experiments were performed as given in detail [48]. Fungal genomic DNA was prepared from plates using the ZR Fungal/Bacterial DNA MiniPrep TM (Zymo Research) kit. 700 ng of isolated genomic DNA was used for restriction enzyme digestion. The southern hybridization was performed with non-radioactive probes by using DIG labeling (Roche) as described in the user protocol.

### Phenotypic assays

The phenotypes and quantification of sporulation for WT and deletion strains were examined as follows: Fungal spores were counted using a haemocytometer. 5 x10^3^ spores (5μl) were used to point inoculate solid GMM, containing appropriate supplements. Plates were incubated in the light (for asexual development) and in the dark (for sexual development) for 4–5 days at 37°C. Colonies were observed using the Olympus szx16 microscope with Olympus sc30 camera. Digital pictures were taken and processed with the Cell Sens Standard software (Olympus). Quantifications were performed from three independent biological replicates.

### Protein extraction

Fungal mycelia were obtained from liquid cultures and broken using liquid nitrogen. Protein extracts were prepared by re-suspending the smooth mycelia in protein extraction buffer (B300 buffer) which contains 300 mM NaCl, 100 mM Tris-Cl pH: 7.5, 10% Glycerol, 1 mM EDTA, 0.1% NP-40 and added with 1mM DTT, 1x Protease inhibitor mix (Roche), 1.5mM Benzamidine, 1x Phosphatase inhibitor mix (Roche) and 1mM PMSF. Protein concentrations were calculated by performing Bradford assays. 100 μg of total protein extract was run on various percentages SDS gels as required (10% or 12%) and transferred to protean membrane with 0.45μm pore size (GE Healthcare).

### Immunoblotting

Antibody conditions and dilutions: For TAP, the primary rabbit α-calmodulin binding peptide (CBP) antibody (05–932, Millipore) as a 1:1000 dilution in TBS with 5% milk. The secondary antibody (goat α-rabbit, G-21234, Life technologies) as a 1:1000 dilution in TBS and 5% milk. For GFP, the primary α-GFP (SC-9996, Santa Cruz) as a 1:500 dilution in TBST with 5% milk. The secondary goat α-mouse (170–6516, Biorad) as a 1:1000 dilution in TBST and 5% milk. For detection of SkpA, custom made rabbit α-SkpA 1:2000 dilutions in TBST and 5% milk (Genscript).

### Tandem Affinity Purification (TAP), GFP pull-downs and LC-MS/MS Protein identification

TAP experiments, GFP pull-downs and preparation of the protein crude extracts and analysis of the proteins were performed as explained in detail [23, 49]. TAP experiments were performed from two independent biological replicates. TAP of the WT strain eluates were used as non-specific control. Proteins identified from StrA, SipA, SipB, SipC, SipD and SipE TAP experiments were filtered from non-specific control purifications. The final lists of the proteins were presented in excel format in Supplementary tables (S1–S6 and S8–S18 Tables). GFP pull-downs of MpkB interaction partners in the presence or absence of StrA originates from either one or two biological replicates (S19 and S20 Tables).

The mass spectrometry proteomics data have been deposited to the ProteomeXchange Consortium (http://proteomecentral.proteomexchange.org/cgi/GetDataset) via the PRIDE partner repository [50] with the dataset identifier PXD011927.

### Confocal microscopy

Green and monomeric red fluorescent protein (GFP and mRFP) expressing strains were grown in 500 μl liquid GMM media, with appropriate supplements in sterile Lab-Tek Chambered Coverglasses with covers (8 wells per coverglass) (Thermo Scientific) for 17–20 hours at 30°C. Localizations of the proteins were captured and recorded as published previously [51].

### RP-HPLC analysis of secondary metabolites

The WT and all deletion strains were grown as follows: 5x10^3^ spores were inoculated on GMM supplemented with vitamins and 1% Oatmeal at 37°C for 5 days. Metabolites were extracted by using chloroform and drying them in speed vac. Samples were suspended in 200μl Methanol. 1mg/ml sterigmatocystin (Sigma) was used as the standard (2.5 μl of standard added in 47.5 μl of 100% Methanol). RP-HPLC analysis was carried out on a Shimadzu RP-HPLC with a photodiode array detector (PDA). 20 μl of standard or sample was injected onto a Luna omega 5μm polar C18 (LC column 150 x 4.6 mm) and separated across a water: acetonitrile gradient with 0.1% (v/v) TriFluoroacetic Acid (TFA). Gradient conditions of 5–100% acetonitrile over 30 min with a flow rate of 1 ml/min were used with PDA detection at 254 nm.

### Quantitative real time PCR

100 mg of mycelia was used for RNA isolation by using RNeasy (Qiagen). 1 μg RNA was used for each cDNA synthesis using the Transcriptor First Strand cDNA Synthesis Kit (Roche). Different primers of target genes were used for qPCR reaction as described in the user protocol, by using LightCycler 480 Sybr Green I Master (Roche). A house-keeping gene, *benA* was used as a standard. Relative Expression Analysis was performed by Light Cycler 480 Software.

### Statistical analysis

All experiments were performed on three independent occasions and numerical data (S1 Dataset) are expressed as the mean ± SD and standard error. The corresponding means were compared for significant differences via the student t-test and One-way ANOVA methods, by using the software Graphpad Prism Version 6.

## Supporting information

S1 Fig Expression pattern and subcellular localization of StrA. (A) Comparative growth and development of *strA*Δ, complementation (StrA-GFP, StrA-TAP) and WT fungal strains. 5x10^3^ fungal spores were point inoculated on glucose minimal medium (GMM) plates for 5 days at 37°C under constant light and dark conditions (only dark is shown here). The central square shows radial growth of colonies and small squares show the stereomicroscopic close-up pictures of the colonies. (B) Quantification of asexual spores and sexual fruit bodies from A. Values are the means of three replicates, and vertical bars represent standard errors. ***Represent values with significant difference in comparison to WT (P < 0.001). (C) Protein expression levels of StrA fused to green fluorescent protein (GFP) during different stages of fungal development. StrA-GFP fusion was monitored in submerged vegetative (20h), on plates in light (asexual for 6, 12, 24h) and in the dark (sexual 6, 12, 24, 48h). 100 μg total protein was applied on each lane. α-GFP detects StrA-GFP fusion, α-SkpA detects SkpA levels used as loading control. Black arrow shows the full length StrA-GFP (116 kDA). Lower bands indicate the degradation products of StrA-GFP. (D) Localization of StrA in living cells. StrA-GFP localized at nuclear envelope. Histone 2A fused to monomeric red fluorescent protein (mRFP) marks the position of the nuclei.
(TIF)

S2 Fig Comparison of alignment of SIKE domain proteins in human and *A. nidulans*. **(**A) Prediction of SIKE domains for *H*. *sapiens* suppressor of IKBKE1 isoform 1 (NP_001095866.1), isoform 2 (NP_079349.2) and *A*. *nidulans* SipB (AN1010) with motif search tool https://www.genome.jp/tools/motif/. Residues shown in red represent SIKE domains. *A*. *nidulans* SipB contains a SIKE domain at its C-terminus. (B) Local alignment of *H*. *sapiens* SIKE isoform 1 and 2 with SIKE domain of *A*. *nidulans* SipB.
(TIF)

S3 Fig Generation of endogenously expressed striatin interacting protein (*sip*) *sgfp* and *ctap* fusions. (A) General depiction of the WT as well as *sgfp* and *ctap* fused *sip* loci. Scale bar (2000 base pairs, bp), restriction enzymes, probes used for Southern hybridizations are shown in the graph. A small proportion of 5′ untranslated region (5′UTR) were used as Southern probes (red bars). (B) Southern hybridizations of *sip-sgfp* and *ctap* fused loci. M: Molecular marker in kilo base pairs (kbp). Sizes of the bands are in line with theoretical maps shown in A. (C) Growth tests of the *sip-sgfp* and *ctap* fusions in comparison to the WT. Tagged strains behave similar to untagged strain (WT) and different from deletions (Fig 2), indicating functionality of the fusions. 5x10^3^ fungal spores were point inoculated on solid GMM plates and incubated for 5 days at 37°C under constant light.
(TIF)

S4 Fig Generation of single and double deletion combinations of *sip* genes. (A) General depiction of the WT *sipA* to *sipE* (*ppgA*) deletion loci. Scale bar (1000 bp), restriction enzymes, probes used for Southern hybridizations are shown in the graph. A small proportion of 5 untranslated region (5′UTR) or 3′UTR were used as Southern probes (red bars). Deletions were created with both *AfpyrG* and *AfpyroA* markers. (B) Southern hybridizations of *sip* single deletions with both marker combinations. Southern hybridizations confirm the replacement of endogenous loci by either *AfpyrG* or *AfpyroA* markers. M: Molecular marker in kbp, T1&T2; Transformant 1&2, respectively. Sizes of the bands are in agreement with theoretical maps shown in A. (C) Southern hybridizations of *strA/sip* and *sip/sip* double deletions. The Southerns in the upper panel show all double deletion combinations by use of either 5′UTR or 3′UTR probes. (D) Lack of open reading frames (ORFs) of the corresponding genes in double deletions. The Southern hybridizations display the lack of respective ORFs of *sipA* to *sipE* in double deletion combinations. Respective ORFs of *sipA* to *sipE* were used as the Southern probe in the Southern hybridizations.
(TIF)

S5 Fig Growth responses of the STRIPAK complex mutants in the presence of DNA damaging stressors. (A) Sensitivity tests of STRIPAK mutants and their complementation in Hydroxyurea (HU, 5.2 mM). (B) Sensitivity tests in Methyl methanesulfonate (MMS, 0.03% mM) and (C) Ethyl methanesulfonate (EMS, 0.01%). The cultures (5x10^3^ spores) were grown for 5 days at 37°C in light. Scale bar 1 cm. These experiments were repeated at least three times. Chart graphs show radial growth diameter compared with WT, which was used as standard (100%). Data are indicated as average ± SD of three independent biological repetitions. Columns with (ns) denote non-significant but (n) denote significant difference and also (***) represent values for strong significant difference (P<0.0001) compared with WT.
(TIF)

S6 Fig Comparison of the radial growth rates of STRIPAK mutants exposed to amino acid starvation and osmotic stress agents. (A) Growth behavior of the STRIPAK mutants in 3-amino 1,2,4 triazole (3-AT, 1 mM) containing GMM media, (B) Caffeine (2 mM) containing media and (C) in osmotic stress NaCl (1 M) media. Strains were grown and statistically analyzed as in S5 and Fig 3.
(TIF)

S1 Table Proteins interacting with StrA during vegetative growth (24 h) at 37°C. (XLSX)

S2 Table Proteins interacting with StrA during asexual growth under light (6 h) at 37°C. (XLSX)

S3 Table Proteins interacting with StrA during asexual growth under light (24 h) at 37°C. (XLSX)

S4 Table Proteins interacting with StrA during sexual growth under dark (6 h) at 37°C. (XLSX)

S5 Table Proteins interacting with StrA during sexual growth under dark (24 h) at 37°C. (XLSX)

S6 Table Proteins interacting with StrA during sexual growth under dark (48 h) at 37°C. (XLSX)

S7 Table Homologs of STRIPAK complex components in eukaryotes. (DOCX)

S8 Table Proteins interacting with SipA during vegetative growth (24 h) at 37°C. (XLSX)

S9 Table Proteins interacting with SipB during vegetative growth (24 h) at 37°C. (XLSX)

S10 Table Proteins interacting with SipC during vegetative growth (24 h) at 37°C. (XLSX)

S11 Table Proteins interacting with SipD during vegetative growth (24 h) at 37°C. (XLSX)

S12 Table Proteins interacting with SipE during vegetative growth (24 h) at 37°C. (XLSX)

S13 Table Comparative interactions of SipA in the presence and absence of StrA during vegetative growth (24 h) at 37°C. (XLSX)

S14 Table Comparative interactions of SipB in the presence and absence of StrA during vegetative growth (24 h) at 37°C. (XLSX)

S15 Table Comparative interactions of SipC in the presence and absence of StrA during vegetative growth (24 h) at 37°C. (XLSX)

S16 Table Comparative interactions of SipD in the presence and absence of StrA during vegetative growth (24 h) at 37°C. (XLSX)

S17 Table Comparative interactions of SipE in the presence and absence of StrA during vegetative growth (24 h) at 37°C. (XLSX)

S18 Table Proteins interacting with StrA in the absence of SipA during vegetative growth (24 h) at 37°C. (XLSX)

S19 Table Interaction partners of MpkB in the presence of StrA during vegetative growth (24 h) at 37°C. (XLSX)

S20 Table Interaction partners of MpkB in the absence of StrA during vegetative growth (24 h) at 37°C. (XLSX)

S21 Table Fungal strains created or used in this study. (DOCX)

S22 Table Plasmids created or used in this study. (DOCX)

S23 Table Oligonucleotides employed in this study. (DOCX)

S1 Dataset Numerical data plotted in graphs. (XLSX)
