# Supplementary figures and images for: Assembly of a heptameric STRIPAK complex is required for coordination of light-dependent multicellular fungal development with secondary metabolism in Aspergillus nidulans
Source: PLoS Genet. 2019 Mar 18;15(3):e1008053. doi: 10.1371/journal.pgen.1008053 (PMC6438568; doi:10.1371/journal.pgen.1008053)

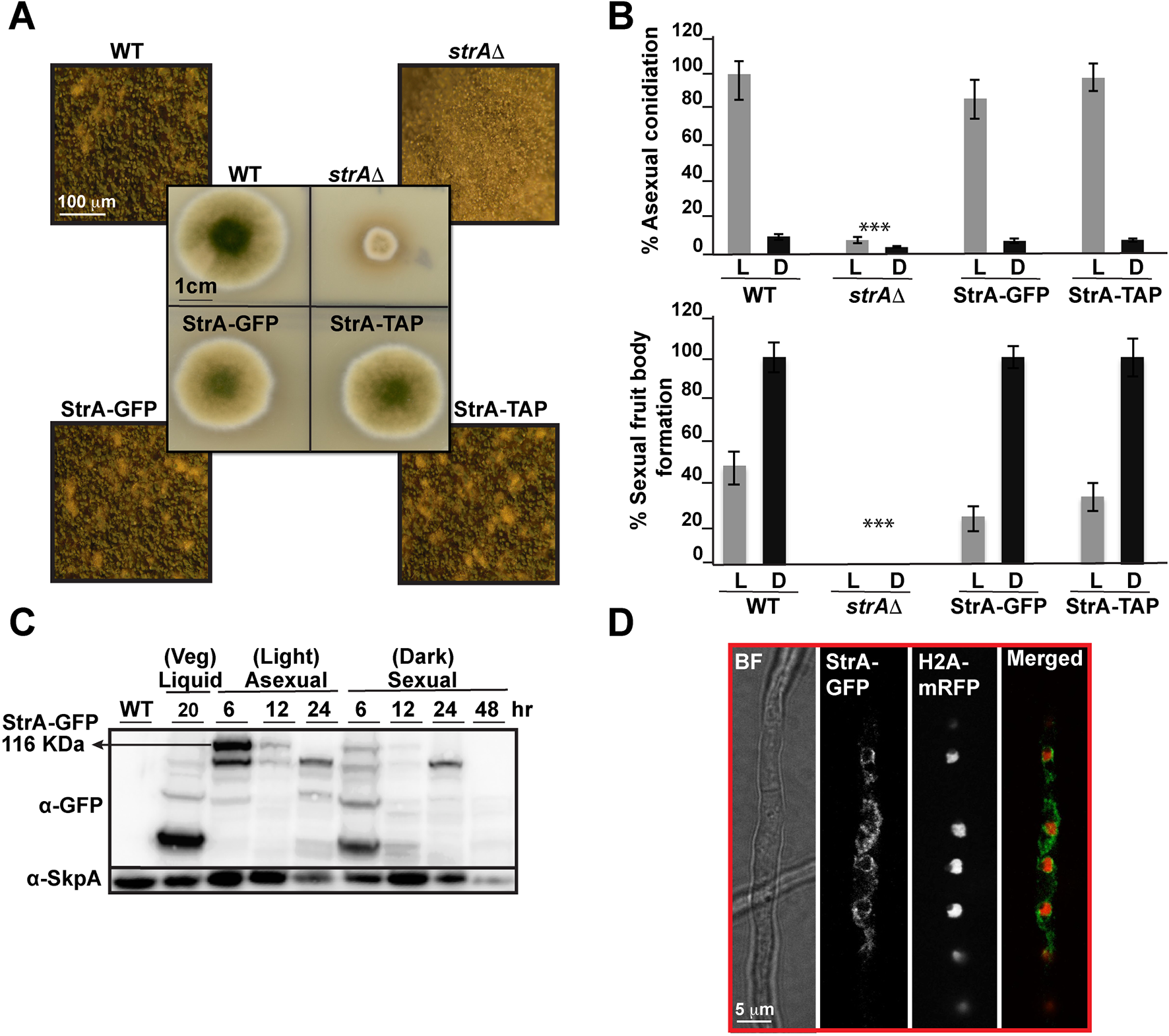

Supplement: S1 Fig — (A) Comparative growth and development of strAΔ, complementation (StrA-GFP, StrA-TAP) and WT fungal strains. 5x10^3^ fungal spores were point inoculated on glucose minimal medium (GMM) plates for 5 days at 37°C under constant light and dark conditions (only dark is shown here). The central square shows radial growth of colonies and small squares show the stereomicroscopic close-up pictures of the colonies. (B) Quantification of asexual spores and sexual fruit bodies from A. Values are the means of three replicates, and vertical bars represent standard errors. ***Represent values with significant difference in comparison to WT (P < 0.001). (C) Protein expression levels of StrA fused to green fluorescent protein (GFP) during different stages of fungal development. StrA-GFP fusion was monitored in submerged vegetative (20h), on plates in light (asexual for 6, 12, 24h) and in the dark (sexual 6, 12, 24, 48h). 100 μg total protein was applied on each lane. α-GFP detects StrA-GFP fusion, α-SkpA detects SkpA levels used as loading control. Black arrow shows the full length StrA-GFP (116 kDA). Lower bands indicate the degradation products of StrA-GFP. (D) Localization of StrA in living cells. StrA-GFP localized at nuclear envelope. Histone 2A fused to monomeric red fluorescent protein (mRFP) marks the position of the nuclei. (TIF) [file pgen.1008053.s001.tif]

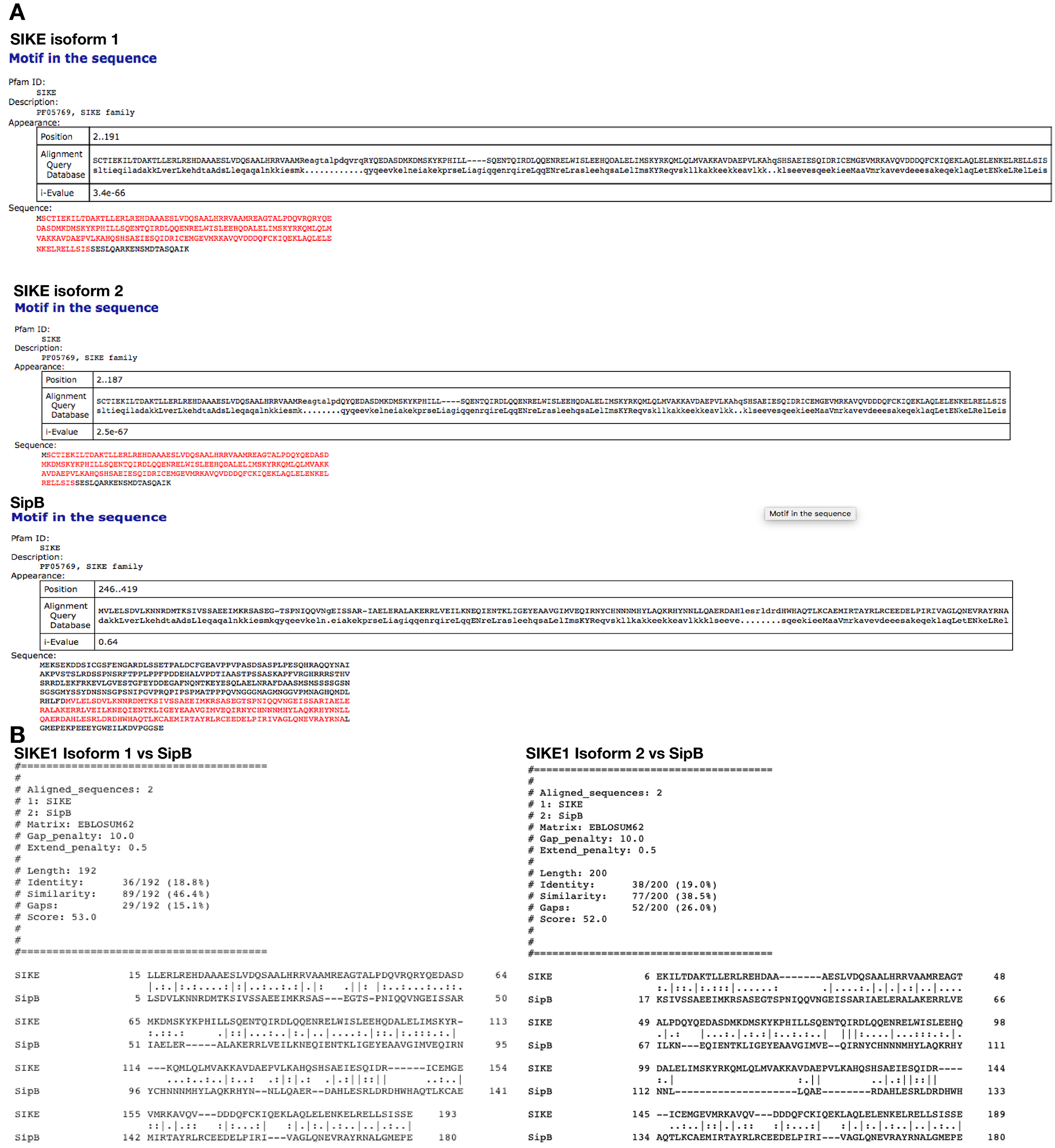

Supplement: S2 Fig — (A) Prediction of SIKE domains for H. sapiens suppressor of IKBKE1 isoform 1 (NP_001095866.1), isoform 2 (NP_079349.2) and A. nidulans SipB (AN1010) with motif search tool https://www.genome.jp/tools/motif/. Residues shown in red represent SIKE domains. A. nidulans SipB contains a SIKE domain at its C-terminus. (B) Local alignment of H. sapiens SIKE isoform 1 and 2 with SIKE domain of A. nidulans SipB. (TIF) [file pgen.1008053.s002.tif]

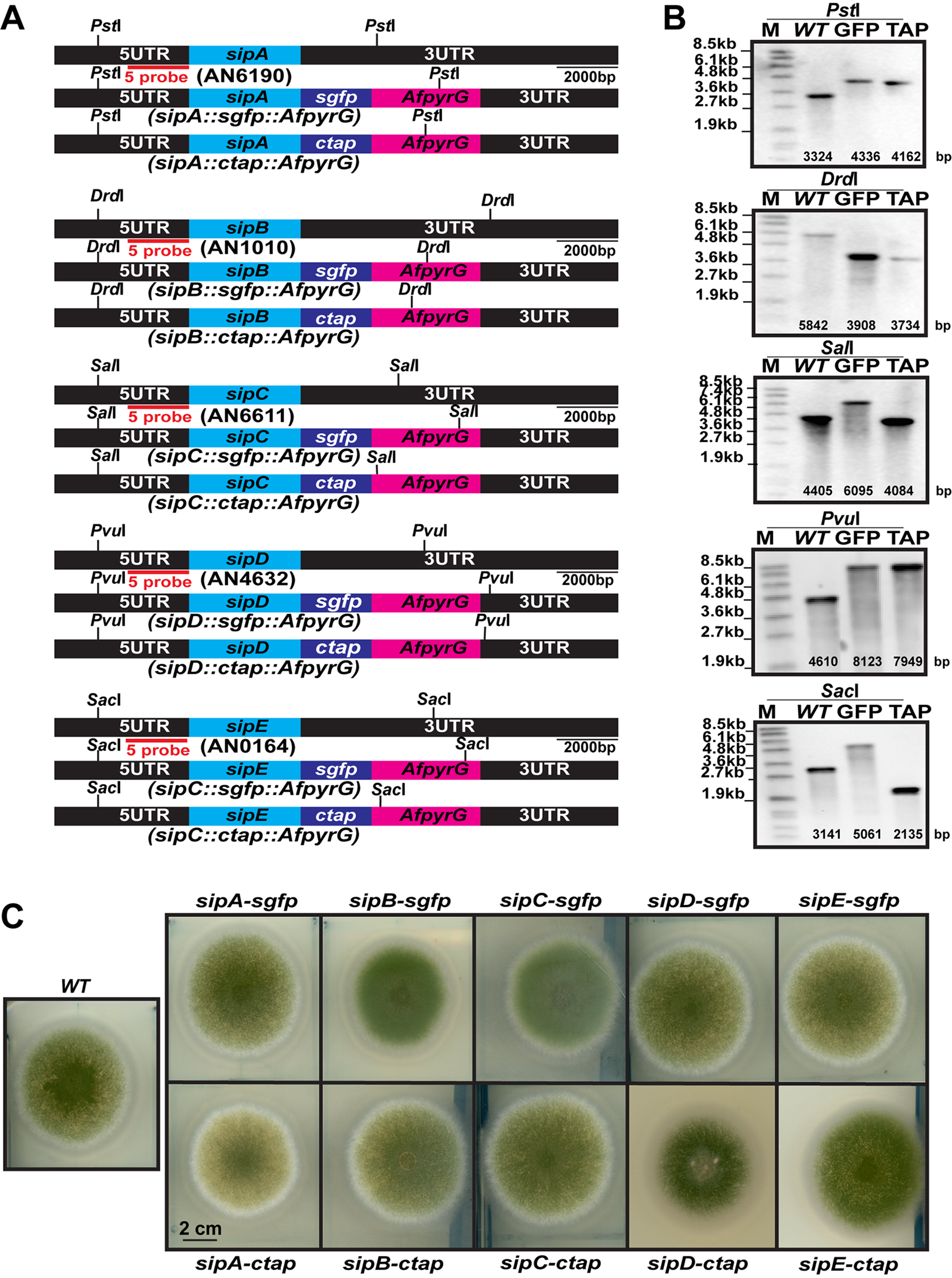

Supplement: S3 Fig — (A) General depiction of the WT as well as sgfp and ctap fused sip loci. Scale bar (2000 base pairs, bp), restriction enzymes, probes used for Southern hybridizations are shown in the graph. A small proportion of 5′ untranslated region (5′UTR) were used as Southern probes (red bars). (B) Southern hybridizations of sip-sgfp and ctap fused loci. M: Molecular marker in kilo base pairs (kbp). Sizes of the bands are in line with theoretical maps shown in A. (C) Growth tests of the sip-sgfp and ctap fusions in comparison to the WT. Tagged strains behave similar to untagged strain (WT) and different from deletions (Fig 2), indicating functionality of the fusions. 5x10^3^ fungal spores were point inoculated on solid GMM plates and incubated for 5 days at 37°C under constant light. (TIF) [file pgen.1008053.s003.tif]

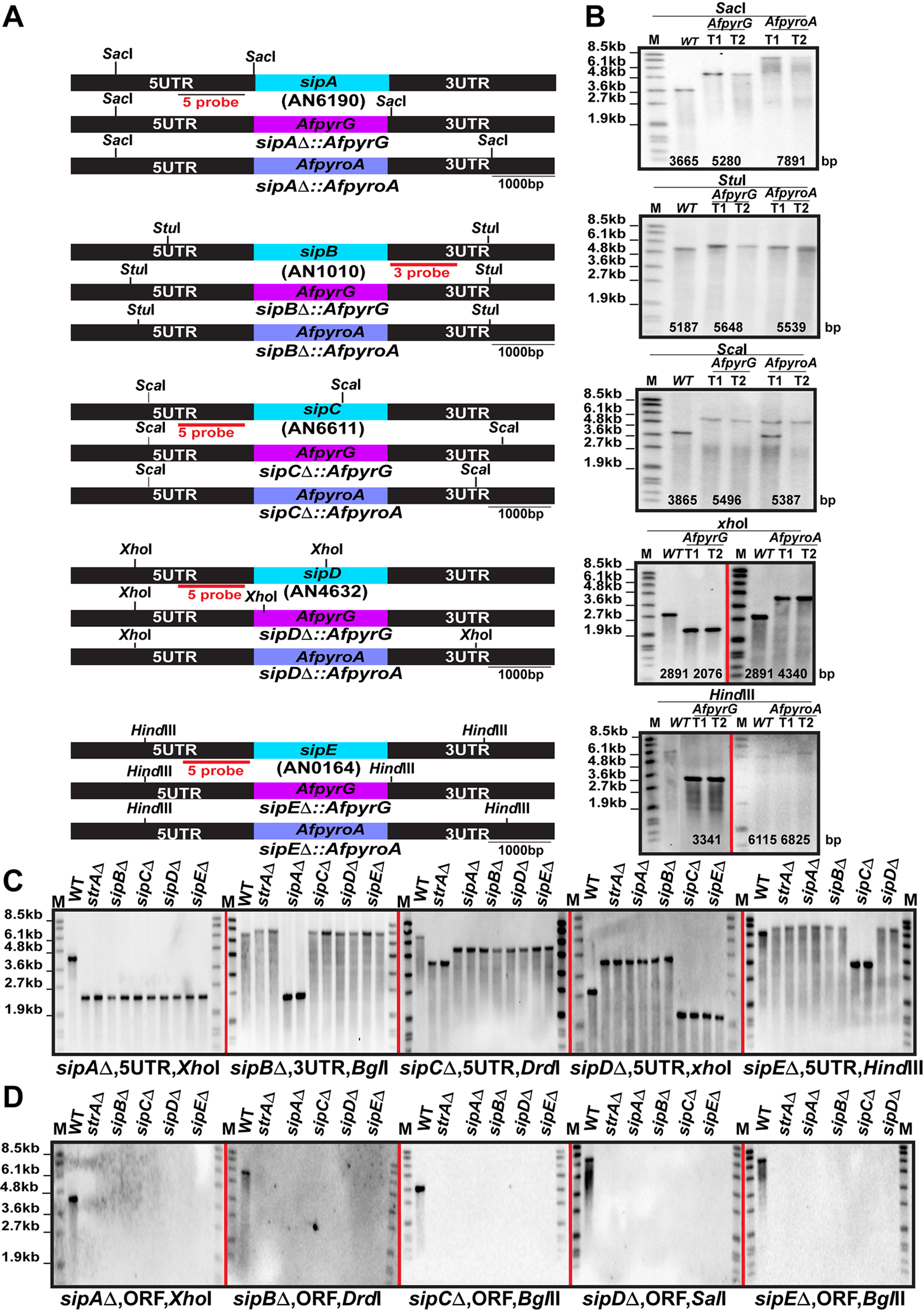

Supplement: S4 Fig — (A) General depiction of the WT sipA to sipE (ppgA) deletion loci. Scale bar (1000 bp), restriction enzymes, probes used for Southern hybridizations are shown in the graph. A small proportion of 5 untranslated region (5′UTR) or 3′UTR were used as Southern probes (red bars). Deletions were created with both AfpyrG and AfpyroA markers. (B) Southern hybridizations of sip single deletions with both marker combinations. Southern hybridizations confirm the replacement of endogenous loci by either AfpyrG or AfpyroA markers. M: Molecular marker in kbp, T1&T2; Transformant 1&2, respectively. Sizes of the bands are in agreement with theoretical maps shown in A. (C) Southern hybridizations of strA/sip and sip/sip double deletions. The Southerns in the upper panel show all double deletion combinations by use of either 5′UTR or 3′UTR probes. (D) Lack of open reading frames (ORFs) of the corresponding genes in double deletions. The Southern hybridizations display the lack of respective ORFs of sipA to sipE in double deletion combinations. Respective ORFs of sipA to sipE were used as the Southern probe in the Southern hybridizations. (TIF) [file pgen.1008053.s004.tif]

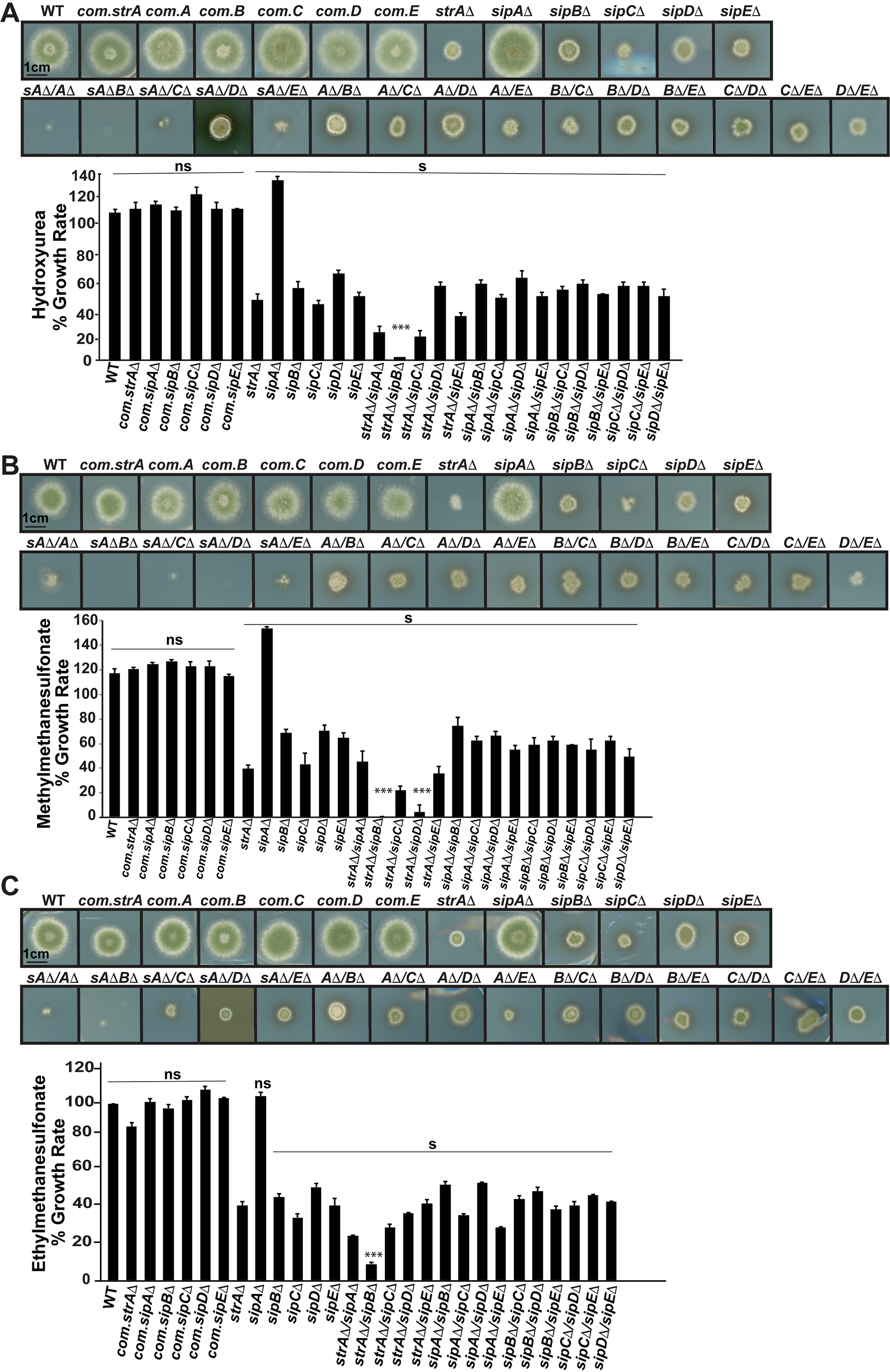

Supplement: S5 Fig — (A) Sensitivity tests of STRIPAK mutants and their complementation in Hydroxyurea (HU, 5.2 mM). (B) Sensitivity tests in Methyl methanesulfonate (MMS, 0.03% mM) and (C) Ethyl methanesulfonate (EMS, 0.01%). The cultures (5x10^3^ spores) were grown for 5 days at 37°C in light. Scale bar 1 cm. These experiments were repeated at least three times. Chart graphs show radial growth diameter compared with WT, which was used as standard (100%). Data are indicated as average ± SD of three independent biological repetitions. Columns with (ns) denote non-significant but (n) denote significant difference and also (***) represent values for strong significant difference (P<0.0001) compared with WT. (TIF) [file pgen.1008053.s005.tif]

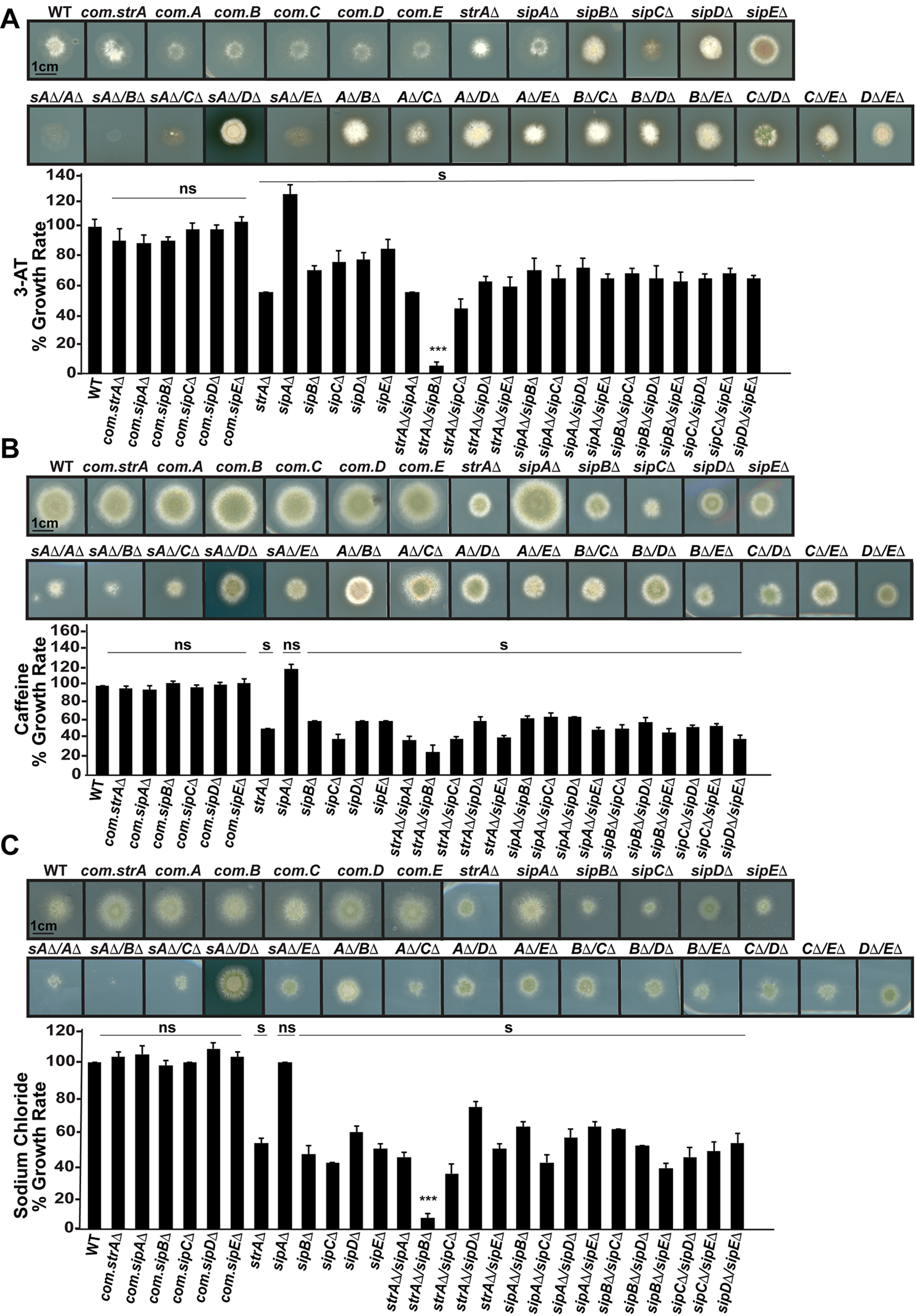

Supplement: S6 Fig — (A) Growth behavior of the STRIPAK mutants in 3-amino 1,2,4 triazole (3-AT, 1 mM) containing GMM media, (B) Caffeine (2 mM) containing media and (C) in osmotic stress NaCl (1 M) media. Strains were grown and statistically analyzed as in S5 and Fig 3. (TIF) [file pgen.1008053.s006.tif]
