# Supplementary material for: Assembly of a heptameric STRIPAK complex is required for coordination of light-dependent multicellular fungal development with secondary metabolism in Aspergillus nidulans
Source: PLoS Genet. 2019 Mar 18;15(3):e1008053. doi: 10.1371/journal.pgen.1008053 (PMC6438568; doi:10.1371/journal.pgen.1008053)
Supplement: S7 Table — (DOCX) [file pgen.1008053.s013.docx]

| *Homo Sapiens* | *Drosophila*  *melanogaster* | *Aspergillus nidulans* | *Neurospora*  *crassa* | *Sordaria macrospora* | *Saccharomyces cerevisiae* | *Schizosaccharomyces pombe* |
| --- | --- | --- | --- | --- | --- | --- |
| STRN1/3/4  (NP_003153)  (NP_001077362)  (XP_024307244) | Cka  (NP_001245936) | StrA  (AN8071) | HAM-3  (XP_963602) | PRO11  (XP_003345440) | Far8  (NP_013742) | Csc3, Far8  (NP_595116) |
| PP2AA  (3DW8_A) | Pp2A-29B  (NP_001245944) | SipF  (AN4085) | PP2A-A  (XP_001728513) | PP2AA  (XP_003348897) | TPD3  (NP_009386) | Paa1  (NP_594948) |
| PP2Ac  (NP_001341948) | mts  (NP_476805) | SipE  (PpgA, AN5791) | PP2-A  (XP_961818) | PP2Ac1  (XP_003346505) | Ppg1  (NP_014429) | Ppa3  (NP_593740) |
| STRIP1/2  (NP_149079)  (NP_065755) | SipA/B  (NP_728867)  (NP_647806) | SipC  (AN6611) | HAM-2  (XP_011394573) | Pro22  (XP_003352145) | Far11  (NP_014272) | Csc2  (NP_595536) |
| MOB4  (NP_056202) | Mob4  (NP_610229) | SipA  (AN6190) | Mob1-2/Mob-3  (XP_962316) | SmMOB3  (CBN80575) | - | - |
| SLMAP  (AAI14628) | SLMAP  (NP_001036399) | SipD  (AN4632) | HAM-4  (XP_964080) | PRO45  (XP_003352390) | Far9/10  (NP_010486)  (NP_013339) | Far10/Csc1  (CAA20309) |
| MST4  (3GGF_A) | GCKIII  (NP_650596) | - | - | SMKIN3/24  (XP_024511144) | - | - |
| Ccm3  (NP_009148) | Ccm3  (NP_650459) | - | - | - | - | - |
| SIKE1  (NP_079349) | Fgop2  (NP_609084) | SipB  (AN1010) | NCU04324  (EAA31734) | SCI1  (XP_003349276) | - | - |

**S7 Table**. Homologs of STRIPAK complex components in eukaryotes
