# Supplementary material for: Assembly of a heptameric STRIPAK complex is required for coordination of light-dependent multicellular fungal development with secondary metabolism in Aspergillus nidulans
Source: PLoS Genet. 2019 Mar 18;15(3):e1008053. doi: 10.1371/journal.pgen.1008053 (PMC6438568; doi:10.1371/journal.pgen.1008053)
Supplement: S21 Table — (DOCX) [file pgen.1008053.s027.docx]

| **Table S21.** Fungal strains created or used in this study | |  |  |
| --- | --- | --- | --- |
| **Strain** | **Genotype** | **Plasmid used** | **Reference** |
| AGB551 | *nkuA*Δ*::argB,pyrG89,pyroA4,veA+* | not applied | [1] |
| AGB655 | *pgpdA::mrfp::h2A-pyrG; mpkB::sgfp::natR; nkuA***∆***::argB*, *pyrG89, pyroA4, veA+* | not applied | [1] |
| StrA-GFP-RFP | *gpdA::mrfp::h2A::pyroA;strA::sgfp::natR;nkuA*Δ*::argB,pyrG89,pyroA4,veA+* | pOB340 in StrA-GFP | This study |
| StrA-GFP | *strA::sgfp::natR;nkuA*Δ*::argB,pyrG89,pyroA4,veA+* | pOB480 in AGB551 | This study |
| StrA-TAP | *strA::ctap::natR;nkuA*Δ*::argB,pyrG89,pyroA4,veA+* | pOB481 in AGB551 | This study |
| StrA-DEL1 | *strA*Δ*::prtA;nkuA*Δ*::argB,pyrG89,pyroA4,veA+* | pOB525 in AGB551 | This study |
| StrA-DEL2 | *strA*Δ*::AfpyroA;nkuA*Δ*::argB,pyrG89,pyroA4,veA+* | pOB526 in AGB551 | This study |
| StrA-mRFP | *strA::mrfp::AfpyrG;nkuA*Δ*::argB,pyrG89,pyroA4,veA+* | pOB527 in AGB551 | This study |
| ANNE1.1 | *sipA*Δ*::AfpyrG;nkuA*Δ*::argB,pyrG89,pyroA4,veA+* | pNE1 in AGB551 | This study |
| ANNE1.2 | *sipA*Δ*::AfpyroA;nkuA*Δ*::argB,pyrG89,pyroA4,veA+* | pNE2 in AGB551 | This study |
| ANNE2.1 | *sipB*Δ*::AfpyrG;nkuA*Δ*::argB,pyrG89,pyroA4,veA+* | pNE3 in AGB551 | This study |
| ANNE2.2 | *sipB*Δ*::AfpyroA;nkuA*Δ*::argB,pyrG89,pyroA4,veA+* | pNE4 in AGB551 | This study |
| ANNE3.1 | *sipC*Δ*::AfpyrG;nkuA*Δ*::argB,pyrG89,pyroA4,veA+* | pNE5 in AGB551 | This study |
| ANNE3.2 | *sipC*Δ*::AfpyroA;nkuA*Δ*::argB,pyrG89,pyroA4,veA+* | pNE6 in AGB551 | This study |
| ANNE4.1 | *sipDΔ::AfpyrG;nkuAΔ::argB,pyrG89,pyroA4,veA+* | pNE9 in AGB551 | This study |
| ANNE4.2 | *sipD*Δ*::AfpyroA;nkuA*Δ*::argB,pyrG89,pyroA4,veA+* | pNE10 in AGB551 | This study |
| ANNE5.1 | *sipE*Δ*::AfpyrG;nkuA*Δ*::argB,pyrG89,pyroA4,veA+* | pNE11 in AGB551 | This study |
| ANNE5.2 | *sipE*Δ*::AfpyroA;nkuA*Δ*::argB,pyrG89,pyroA4,veA+* | pNE12 in AGB551 | This study |
| ANNE1.3 | *sipA*Δ*::AfpyrG;strA*Δ*::ptrA;nkuA*Δ*::argB,pyrG89,pyroA4,veA+* | pNE1 in StrA-DEL1 | This study |
| ANNE2.3 | *sipB*Δ*::AfpyrG;strA*Δ*::ptrA;nkuA*Δ*::argB,pyrG89,pyroA4,veA+* | pNE3 in StrA-DEL1 | This study |
| ANNE3.3 | *sipC*Δ*::AfpyrG;strA*Δ*::ptrA;nkuA*Δ*::argB,pyrG89,pyroA4,veA+* | pNE5 in StrA-DEL1 | This study |
| ANNE4.3 | *sipD*Δ*::AfpyroA;strA*Δ*::ptrA;nkuA*Δ*::argB,pyrG89,pyroA4,veA+* | pNE9 in StrA-DEL1 | This study |
| ANNE5.3 | *sipE*Δ*::AfpyroA;strA*Δ*::ptrA;nkuA*Δ*::argB,pyrG89,pyroA4,veA+* | pNE11 in StrA-DEL1 | This study |
| ANNE6 | *sipA*Δ*::AfpyrG;sipB*Δ*::AfpyroA;nkuA*Δ*::argB,pyrG89,pyroA4,veA+* | pNE4 in ANNE1.1 | This study |
| ANNE7 | *sipA*Δ*::AfpyrG;sipC*Δ*::AfpyroA;nkuA*Δ*::argB,pyrG89,pyroA4,veA+* | pNE6 inANNE1.1 | This study |
| ANNE8 | *sipA*Δ*::AfpyrG;sipD*Δ*::AfpyroA;nkuA*Δ*::argB,pyrG89,pyroA4,veA+* | pNE10 in ANNE1.1 | This study |
| ANNE9 | *sipA*Δ*::AfpyrG;sipE*Δ*::AfpyroA;nkuA*Δ*::argB,pyrG89,pyroA4,veA+* | pNE12 in ANNE1.1 | This study |
| ANNE10 | *sipB*Δ*::AfpyroA;sipC*Δ*::AfpyrG;nkuA*Δ*::argB,pyrG89,pyroA4,veA+* | pNE4 in ANNE3.1 | This study |
| ANNE11 | *sipB*Δ*::AfpyroA;sipD*Δ*::AfpyG;nkuA*Δ*::argB,pyrG89,pyroA4,veA+* | pNE4 in ANNE4.1 | This study |
| ANNE12 | *sipB*Δ*::AfpyroA;sipE*Δ*::AfpyrG;nkuA*Δ*::argB,pyrG89,pyroA4,veA+* | pNE4 in ANNE5.1 | This study |
| ANNE13 | *sipC*Δ*::AfpyroA;sipD*Δ*::AfpyrG;nkuA*Δ*::argB,pyrG89,pyroA4,veA+* | pNE6 in ANNE4.1 | This study |
| ANNE14 | *sipC*Δ*::AfpyroA;sipE*Δ*::AfpyrG;nkuA*Δ*::argB,pyrG89,pyroA4,veA+* | pNE6 in ANNE5.1 | This study |
| ANNE15 | *sipD*Δ*::AfpyroA;sipE*Δ*::AfpyrG;nkuA*Δ*::argB,pyrG89,pyroA4,veA+* | pNE10 in ANNE5.1 | This study |
| ANNE16 | *sipA::sgfp::AfpyrG;nkuA*Δ*::argB,pyrG89,pyroA4,veA+* | pNE13 in AGB551 | This study |
| ANNE17 | *sipB::sgfp::AfpyrG;nkuA*Δ*::argB,pyrG89,pyroA4,veA+* | pNE15 in AGB551 | This study |
| ANNE18 | *sipC::sgfp::AfpyrG;nkuA*Δ*::argB,pyrG89,pyroA4,veA+* | pNE17 in AGB551 | This study |
| ANNE19 | *sipD::sgfp::AfpyrG;nkuA*Δ*::argB,pyrG89,pyroA4,veA+* | pNE19 in AGB551 | This study |
| ANNE20 | *sipE::sgfp::AfpyrG;nkuA*Δ*::argB,pyrG89,pyroA4,veA+* | pNE21 in AGB551 | This study |
| ANNE21 | *sipA::ctap::AfpyrG;nkuA*Δ*::argB,pyrG89,pyroA4,veA+* | pNE14 in AGB551 | This study |
| ANNE22 | *sipB::ctap::AfpyrG;nkuA*Δ*::argB,pyrG89,pyroA4,veA+* | pNE16 in AGB551 | This study |
| ANNE23 | *sipC::ctap::AfpyrG;nkuA*Δ*::argB,pyrG89,pyroA4,veA+* | pNE18 in AGB551 | This study |
| ANNE24 | *sipD::ctap::AfpyrG;nkuA*Δ*::argB,pyrG89,pyroA4,veA+* | pNE20 in AGB551 | This study |
| ANNE25 | *sipE::ctap::AfpyrG;nkuA*Δ*::argB,pyrG89,pyroA4,veA+* | pNE22 in AGB551 | This study |
| ANNE26 | *sipA::sgfp::AfpyroA;nkuA*Δ*::argB,pyrG89,pyroA4,veA+* | pNE23 in AGB551 | This study |
| ANNE27 | *sipB::sgfp::AfpyroA;nkuA*Δ*::argB,pyrG89,pyroA4,veA+* | pNE24 in AGB551 | This study |
| ANNE28 | *sipC::sgfp::AfpyroA;nkuA*Δ*::argB,pyrG89,pyroA4,veA+* | pNE25 in AGB551 | This study |
| ANNE29 | *sipD::sgfp::AfpyroA;nkuA*Δ*::argB,pyrG89,pyroA4,veA+* | pNE26 in AGB551 | This study |
| ANNE30 | *sipE::sgfp::AfpyroA;nkuA*Δ*::argB,pyrG89,pyroA4,veA+* | pNE27 in AGB551 | This study |
| ANNE31 | *strA*Δ*::ptrA;sipA::ctap::AfpyrG;nkuA*Δ*::argB,pyrG89,pyroA4,veA+* | pOB525 in ANNE21 | This study |
| ANNE32 | *strA*Δ*::ptrA;sipB::ctap::AfpyrG;nkuA*Δ*::argB,pyrG89,pyroA4,veA+* | pOB525 in ANNE22 | This study |
| ANNE33 | *strA*Δ*::ptrA;sipC::ctap::AfpyrG;nkuA*Δ*::argB,pyrG89,pyroA4,veA+* | pOB525 in ANNE23 | This study |
| ANNE34 | *strA*Δ*::ptrA;sipD::ctap::AfpyrG;nkuA*Δ*::argB,pyrG89,pyroA4,veA+* | pOB525 in ANNE24 | This study |
| ANNE35 | *strA*Δ*::ptrA;sipE::ctap::AfpyrG;nkuA*Δ*::argB,pyrG89,pyroA4,veA+* | pOB525 in ANNE25 | This study |
| ANNE36 | *sipA::sgfp::AfpyroA;strA::mrfp::AfpyrG;nkuA*Δ*::argB,pyrG89,pyroA4,veA+* | pOB527 in ANNE26 | This study |
| ANNE37 | *sipB::sgfp::AfpyroA;strA::mrfp::AfpyrG;nkuA*Δ*::argB,pyrG89,pyroA4,veA+* | pOB527 in ANNE27 | This study |
| ANNE38 | *sipC::sgfp::AfpyroA;strA::mrfp::AfpyrG;nkuA*Δ*::argB,pyrG89,pyroA4,veA+* | pOB527 in ANNE28 | This study |
| ANNE39 | *sipD::sgfp::AfpyroA;strA::mrfp::AfpyrG;nkuA*Δ*::argB,pyrG89,pyroA4,veA+* | pOB527 in ANNE29 | This study |
| ANNE40 | *sipE::sgfp::AfpyroA;strA::mrfp::AfpyrG;nkuA*Δ*::argB,pyrG89,pyroA4,veA+* | pOB527 in ANNE30 | This study |
| ANNE41 | *^p^sipA::sipA::sipA^t^-AfpyroA;sipA*Δ*::AfpyrG;nkuA*Δ*::argB,pyrG89,pyroA4,veA+* | pNE28 in ANNE1.1 | This study |
| ANNE42 | *^p^sipB::sipB::sipB^t^-AfpyroA;sipB*Δ*::AfpyrG;nkuA*Δ*::argB,pyrG89,pyroA4,veA+* | pNE29 in ANNE2.1 | This study |
| ANNE43 | *^p^sipC::sipC::sipC^t^:AfpyroA;sipC*Δ*::AfpyrG;nkuA*Δ*::argB,pyrG89,pyroA4,veA+* | pNE30 in ANNE3.1 | This study |
| ANNE44 | *^p^sipD::sipD::sipD^t^:AfpyroA;sipD*Δ*::AfpyrG;nkuA*Δ*::argB,pyrG89,pyroA4,veA+* | pNE31 in ANNE4.1 | This study |
| ANNE45 | *^p^sipE::sipE::sipE^t^-AfpyroA;sipE*Δ*::AfpyrG;nkuA*Δ*::argB,pyrG89,pyroA4,veA+* | pNE32 in ANNE5.1 | This study |
| ANNE46 | *^p^strA::strA::strA^t^:AfpyroA;strA*Δ*::ptrA;nkuA*Δ*::argB,pyrG89,pyroA4,veA+* | pNE33 in StrA-DEL1 | This study |
| ANNE47 | *^p^gpdA::mrfp::h2A::AfpyroA;sipA::sgfp::AfpyrG;nkuA*Δ*::argB,pyrG89,pyroA4,veA+* | pOB340 in ANNE16 | This study |
| ANNE48 | *^p^gpdA::mrfp::h2A::AfpyroA;sipB::sgfp::AfpyrG;nkuA*Δ*::argB,pyrG89,pyroA4,veA+* | pOB340 in ANNE17 | This study |
| ANNE49 | *^p^gpdA::mrfp::h2A::AfpyroA;sipC::sgfp::AfpyrG;nkuA*Δ*::argB,pyrG89,pyroA4,veA+* | pOB340 in ANNE18 | This study |
| ANNE50 | *^p^gpdA::mrfp::h2A::AfpyroA;sipD::sgfp::AfpyrG;nkuA*Δ*::argB,pyrG89,pyroA4,veA+* | pOB340 in ANNE19 | This study |
| ANNE51 | *^p^gpdA::mrfp::h2A::AfpyroA;sipE::sgfp::AfpyrG;nkuA*Δ*::argB,pyrG89,pyroA4,veA+* | pOB340 in ANNE20 | This study |
| ANNE57 | *^p^gpdA::mrfp::h2A::phlR;sipA::sgfp::AfpyrG;nkuA*Δ*::argB,pyrG89,pyroA4,veA+* | pME3857 in ANNE16 | This study |
| ANNE58 | *^p^gpdA::mrfp::h2A::phlR;sipB::sgfp::AfpyrG;nkuA*Δ*::argB,pyrG89,pyroA4,veA+* | pME3857 in ANNE17 | This study |
| ANNE59 | *^p^gpdA::mrfp::h2A::phlR;sipC::sgfp::AfpyrG;nkuA*Δ*::argB,pyrG89,pyroA4,veA+* | pME3857 in ANNE18 | This study |
| ANNE60 | *^p^gpdA::mrfp::h2A::phlR;sipD::sgfp::AfpyrG;nkuA*Δ*::argB,pyrG89,pyroA4,veA+* | pME3857 in ANNE19 | This study |
| ANNE61 | *^p^gpdA::mrfp::h2A::phlR;sipE::sgfp::AfpyrG;nkuA*Δ*::argB,pyrG89,pyroA4,veA+* | pME3857 in ANNE20 | This study |
| ANNE62 | *strA*Δ*::AfpyroA;pgpdA::mrfp::h2A::phlR;sipA::sgfp::AfpyrG;nkuA*Δ*::argB,pyrG89,pyroA4,veA+* | pOB526 in ANNE57 | This study |
| ANNE63 | *strA*Δ*::AfpyroA;pgpdA::mrfp::h2A::phlR;sipB::sgfp::AfpyrG;nkuA*Δ*::argB,pyrG89,pyroA4,veA+* | pOB526 in ANNE58 | This study |
| ANNE64 | *strA*Δ*::AfpyroA;pgpdA::mrfp::h2A::phlR;sipC::sgfp::AfpyrG;nkuA*Δ*::argB,pyrG89,pyroA4,veA+* | pOB526 in ANNE59 | This study |
| ANNE65 | *strA*Δ*::AfpyroA;pgpdA::mrfp::h2A::phlR;sipD::sgfp::AfpyrG;nkuA*Δ*::argB,pyrG89,pyroA4,veA+* | pOB526 in ANNE60 | This study |
| ANNE66 | *strA*Δ*::AfpyroA;pgpdA::mrfp::h2A::phlR;sipE::sgfp::AfpyrG;nkuA*Δ*::argB,pyrG89,pyroA4,veA+* | pOB526 in ANNE61 | This study |
| ANNE67 | *mpkA::sgfp::AfpyrG; nkuA***∆***::argB*, *pyrG89, pyroA4, veA+* | pBK125 in  AGB551 | This study |
| ANNE68 | *mpkA::sgfp::AfpyrG; nkuA***∆***::argB*, *pyrG89, pyroA4, veA+* | pBK126 in  AGB551 | This study |
| ANNE73 | *strA*Δ*::AfpyroA; pgpdA::mrfp::h2A-pyrG; mpkB::sgfp::natR; nkuA***∆***::argB*, *pyrG89, pyroA4, veA+* | pOB526 in  AGB655 | This study |
| ANBK116.1 | *pgpdA::mrfp::h2A; mpkA::sgfp::AfpyrG; nkuA***∆***::argB*, *pyrG89, pyroA4, veA+* | pME3857 in  ANNE67 | This study |
| ANBK117.2 | *pgpdA::mrfp::h2A; mpkC::sgfp::AfpyrG; nkuA***∆***::argB*, *pyrG89, pyroA4, veA+* | pME3857 in  ANNE68 | This study |
| ANBK119 | *strA*Δ*::AfpyroA; pgpdA::mrfp::h2A::phlR; mpkA::sgfp::AfpyrG; nkuA***∆***::argB*, *pyrG89, pyroA4, veA+* | pOB526 in  ANBK116.1 | This study |
| ANBK120 | *strA*Δ*::AfpyroA; pgpdA::mrfp::h2A::phlR; mpkC::sgfp::AfpyrG; nkuA***∆***::argB*, *pyrG89, pyroA4, veA+* | pOB526 in  ANBK117.2 | This study |

**References**

1. Bayram O, Bayram OS, Ahmed YL, Maruyama J, Valerius O, Rizzoli SO, et al. The *Aspergillus nidulans* MAPK module AnSte11-Ste50-Ste7-Fus3 controls development and secondary metabolism. PLoS Genet. 2012;8(7):e1002816. Epub 2012/07/26. doi: 10.1371/journal.pgen.1002816 PGENETICS-D-11-02521 [pii]. PubMed PMID: 22829779; PubMed Central PMCID: PMC3400554.
