# Supplementary material for: Assembly of a heptameric STRIPAK complex is required for coordination of light-dependent multicellular fungal development with secondary metabolism in Aspergillus nidulans
Source: PLoS Genet. 2019 Mar 18;15(3):e1008053. doi: 10.1371/journal.pgen.1008053 (PMC6438568; doi:10.1371/journal.pgen.1008053)
Supplement: S22 Table — (DOCX) [file pgen.1008053.s028.docx]

| **Table S22.** Plasmids created or used in this study | |  |
| --- | --- | --- |
| **Plasmid** | **Description** | **Reference** |
| pUC19 | *E. coli* cloning plasmid with *bla* (ampicillin resistance gene) gene | Thermo Fisher |
| pME3857 | *pgpdA::mrfp::h2A::trpCt* (histone 2A) with phleomycin (*phlR*) marker | [1] |
| pOSB114 | *Pme*I*::AfpyroA::Swa*I inserted in *Sma*I site of pUC19 (complementation plasmid) | This study |
| pOB340 | *bioA5ORF::AfpyroA::pgpdA::mrfp::bioA3ORF* (histone 2A) with *bla* (*E.coli*) and *AfpyroA* (*A. nidulans*) | This study |
| pOB480 | *strA::sgfp::natR* cassette with *Pme*I site in *Sma*I site of pUC19 | This study |
| pOB481 | *strA::ctap::natR* cassette with *Pme*I site in *Sma*I site of pUC19 | This study |
| pOB525 | *strA* deletion with *ptrA* in *Sma*I site of pUC19 | This study |
| pOB526 | *strA* deletion with *Afpyro*A in *Sma*I site of pUC19 | This study |
| pOB527 | *strA::mrfp* with *AfpyrG* in *Sma*I site of pUC19 | This study |
| pNE1 | *sipA* (AN6190) deletion with *AfpyrG* in *Sma*I site of pUC19 | This study |
| pNE2 | *sipA* (AN6190) deletion with *AfpyroA* in *Sma*I site of pUC19 | This study |
| pNE3 | *sipB* (AN1010) deletion with *AfpyrG* in *Sma*I site of pUC19 | This study |
| pNE4 | *sipB (AN1010)* deletion with *AfpyroA in Sma*I site of pUC19 | This study |
| pNE5 | *sipC* (AN6611) deletion with *AfpyrG* in *Sma*I site of pUC19 | This study |
| pNE6 | *sipC* (AN6611) deletion with *AfpyroA* in *Sma*I site of pUC19 | This study |
| pNE9 | *sipD* (AN4632) deletion with *AfpyrG* in *Sma*I site of pUC19 | This study |
| pNE10 | *sipD* (AN4632) deletion with *AfpyroA* in *Sma*I site of pUC19 | This study |
| pNE11 | *sipE* (AN0164) deletion with *AfpyrG* in *Sma*I site of pUC19 | This study |
| pNE12 | *sipE* (AN0164) deletion with *AfpyroA* in *Sma*I site of pUC19 | This study |
| pNE13 | *sipA* (AN6190) *sgfp::AfpyrG* cassette in *Sma*I site of pUC19 | This study |
| pNE14 | *sipA* (AN6190) *ctap::AfpyrG* cassette in  *Sma*I site of pUC19 | This study |
| pNE15 | *sipB* (AN1010) *sgfp::AfpyrG* cassette *Sma*I site of pUC19 | This study |
| pNE16 | *sipB* (AN1010) *ctap::AfpyrG* cassette in  *Sma*I site of pUC19 | This study |
| pNE17 | *sipC* (AN6611) *sgfp::AfpyrG* cassette in *Sma*I site of pUC19 | This study |
| pNE18 | *sipC* (AN6611) *ctap::AfpyrG* cassette in *Sma*I site of pUC19 | This study |
| pNE19 | *sipD* (AN4632) *sgfp::AfpyrG* cassette in *Sma*I site of pUC19 | This study |
| pNE20 | *sipD* (AN4632) *ctap::AfpyrG* cassette in *Sma*I site of pUC19 | This study |
| pNE21 | *sipE* (AN0164) *sgfp::AfpyrG* cassette in *Sma*I site of pUC19 | This study |
| pNE22 | *sipE* (AN0164) *ctap::AfpyrG* cassette in *Sma*I site of pUC19 | This study |
| pNE23 | *sipA* (AN6190) *sgfp::AfpyroA* cassette in *Sma*I site of pUC19 | This study |
| pNE24 | *sipB* (AN61010) *sgfp::AfpyroA* cassette in *Sma*I site of pUC19 | This study |
| pNE25 | *sipC* (AN6611) *sgfp::AfpyroA* cassette in *Sma*I site of pUC19 | This study |
| pNE26 | *sipD* (AN4632) *sgfp::AfpyroA* cassette in *Sma*I site of pUC19 | This study |
| pNE27 | *sipE* (AN0164) *sgfp::AfpyroA* cassette in  *Sma*I site of pUC19 | This study |
| pNE28 | *sipA* (AN6190) genomic locus in *Pme*I site of the pOSB114 | This study |
| pNE29 | *sipB* (AN1010) genomic locus in *Pme*I site of the pOSB114 | This study |
| pNE30 | *sipC* (AN6611) genomic locus in *Pme*I site of the pOSB114 | This study |
| pNE31 | *sipD* (AN4632) genomic locus in *Pme*I site of the pOSB114 | This study |
| pNE32 | *sipE* (AN0164) genomic locus in *Pme*I site of the pOSB114 | This study |
| pNE33 | *strA* (AN8071) genomic locus in *Pme*I site of the pOSB114 | This study |
| pBK125 | *mpkA*::*sgfp::AfpyrG* cassette in *Sma*I site of pUC19 | This study |
| pBK126 | *mpkC*::*sgfp::AfpyrG* cassette in *Sma*I site of pUC19 | This study |

1. Bayram O, Bayram OS, Ahmed YL, Maruyama J, Valerius O, Rizzoli SO, et al. The *Aspergillus nidulans* MAPK module AnSte11-Ste50-Ste7-Fus3 controls development and secondary metabolism. PLoS Genet. 2012;8(7):e1002816. Epub 2012/07/26. doi: 10.1371/journal.pgen.1002816 PGENETICS-D-11-02521 [pii]. PubMed PMID: 22829779; PubMed Central PMCID: PMC3400554.
