# Supplementary material for: Assembly of a heptameric STRIPAK complex is required for coordination of light-dependent multicellular fungal development with secondary metabolism in Aspergillus nidulans
Source: PLoS Genet. 2019 Mar 18;15(3):e1008053. doi: 10.1371/journal.pgen.1008053 (PMC6438568; doi:10.1371/journal.pgen.1008053)
Supplement: S23 Table — (DOCX) [file pgen.1008053.s029.docx]

| **Table 23.** Oligonucleotides employed in this study | |  |
| --- | --- | --- |
| **Designation** | **Sequence in 5' > 3' direction** | **Size** |
| NE1 (5UTR AN6190 pUC tail) | TTC GAG CTC GGT ACC CGA CAG GAC TGG TCC AGA GTG | 36 mer |
| NE2 (5UTR AN6190 nest) | GTG GTC GTG ATC AGA ACC GTC | 21 mer |
| NE3 (5UTR AN6190 pyrG tail) | GAG CAT TGT TTG AGG CAG TCA GGA AGG TGA ATT TGG GAG | 39 mer |
| NE4 (3UTR AN6190 pyrG/pyroA tail) | GCC TCC TCT CAG ACA GTG TGA CGA ACG GCG CTC AAA C | 37 mer |
| NE5 (3UTR AN6190 pUC tail) | ACT CTA GAG GAT CCC CGT ACTG TCG ACG TTG AAT AGA TAC | 40 mer |
| NE6 (3UTR AN6190 nest) | GCT ATA TCC TCC ACG CAC CTA G | 22 mer |
| NE7 (5UTR AN6190 pyroA tail) | CCA GCA TCT GAT GTC CAG TCA GGA AGG TGA ATT TGG GAG | 39 mer |
| NE8 (5UTR AN1010 pUC tail) | TTC GAG CTC GGT ACC CGT AAG TTC AAT GCA CTC GCC TTG | 39 mer |
| NE9 (5UTR AN1010 nest) | CAA CTA GCG TTG TCG CAT TCA C | 22 mer |
| NE10 (5UTR AN1010 pyrG tail) | GAG CAT TGT TTG AGG CGG TGT TCT ACA GGA ATG TGA GAT G | 40 mer |
| NE11 (3UTR AN1010 pyrG/pyroA tail) | GCC TCC TCT CAG ACA GTG TAG CGC ACG CTG CTG ATA C | 37 mer |
| NE12 (3UTR AN1010 pUC19 tail) | ACT CTA GAG GAT CCC CGT CAG ATG AGG GAG TAG TTG AC | 38 mer |
| NE13 (3UTR AN1010 nest) | GTG TGA TTG GGT TCT CAA GAG G | 22 mer |
| NE14 (5UTR AN1010 pyroA tail) | CCA GCA TCT GAT GTC CGG TGT TCT ACA GGA ATG TGA GAT G | 40 mer |
| NE15 (5UTR AN6611 pUC tail) | TTC GAG CTC GGT ACC CGT AGG CCG CAG AAG GTT ACG | 36 mer |
| NE16 (5UTR AN6611 nest) | GTG CCA CTC GGT CTA GTG TC | 20 mer |
| NE17 (5UTR AN6611 pyrG tail) | GAG CAT TGT TTG AGG CGA TCG GGT ATA GTA ATT AAG AAG GTG | 42 mer |
| NE18 (3UTR AN6611 pyrG/pyroA tail) | GCC TCC TCT CAG ACA GCT ACT TCA TAG TAC AGT TAT GTA CCT C | 43 mer |
| NE19 (3UTR AN6611 pUC tail) | ACT CTA GAG GAT CCC CCT CAT CAC TAT CCT CCT CAT CC | 38 mer |
| NE20 (3UTR AN6611 nest) | TCG TCG GAA TAA TCA TCG CTA GAG | 24 mer |
| NE21 (5UTR AN6611 pyroA tail) | CCA GCA TCT GAT GTC CGA TCG GGT ATA GTA ATT AAG AAG GTG | 42 mer |
| NE22 (5UTR AN4085 pUC tail) | TTC GAG CTC GGT ACC CGT TTC AGA ATA TGG TGC GTA CCG | 39 mer |
| NE23 (5UTR AN4085 nest) | TGA GTT GGG GTG GTT CCT GC | 20 mer |
| NE24 (5UTR AN4085 pyrG tail) | GAG CAT TGT TTG AGG CAC CAG AGG GCA AGG ATG TGA C | 37 mer |
| NE25 (3UTR AN4085 pyrG/pyroA tail) | GCC TCC TCT CAG ACA GGT TGA CAC ACT AGG GCT TGA AG | 38 mer |
| NE26 (3UTR AN4085 pUC tail) | ACT CTA GAG GAT CCC CGC AAC ACA CAG CAA GAC CAC C | 37 mer |
| NE27 (3UTR AN4085 nest) | GAA AGA TGC ACA GCG TAG AAA GC | 23 mer |
| NE28 (5UTR AN4085 pyroA tail) | CCA GCA TCT GAT GTC CAC CAG AGG GCA AGG ATG TGA C | 37 mer |
| NE29 (AN1010 5UTR OUT) | CAG CAG CTT AGA CCA ACT GCC | 21 mer |
| NE30 (AN1010 ORF FWD) | GTG GCT CCT TTG AGA ATG GTG | 21 mer |
| NE31 (AN1010 ORF RVS) | TCC CAG CCG TAC TCT TCC TC | 20 mer |
| NE32 (AN6190 5UTR OUT) | CAG CCA GAC ATC TAC GAG AGG | 21 mer |
| NE33 (AN6190 ORF FWD) | GTT CAC AGA GGT GCA GAT TGG | 21 mer |
| NE34 (AN6190 ORF RVS) | GCT CCT CTT CCT TCT GTC TTG | 21 mer |
| NE35 (AN6611 5UTR OUT) | CCT ACT TCC CCT TAC CTG AGC | 21 mer |
| NE36 (AN6611 ORF FWD) | CAT CCG CTG ATG CTT CCT TGG | 21 mer |
| NE37 (AN6611 ORF RVS) | CTG TAG CAT GCC ATC CCA TTC | 21 mer |
| NE38 (AN4085 5UTR OUT) | GTC ATG AGT ACC AAT GGA GAC G | 22 mer |
| NE39 (AN4085 ORF FWD) | GCT CTA TCC TAT CGC TGT TCT C | 22 mer |
| NE40 (AN4085 ORF RVS) | CCG AAC ATC AAC ATC ATC ATC GC | 23 mer |
| NE41 (AN4632 5UTR pUC tail) | TTC GAG CTC GGT ACC CCG CAC CCA TCT TCA CTG TAG C | 37 mer |
| NE42 (AN4632 5UTR pyrG tail) | GAG CAT TGT TTG AGG CGG TGT TAT GGA TGC TCG ACG C | 37 mer |
| NE43 (AN4632 3UTR pyrG/pyroA tail) | GCC TCC TCT CAG ACA GCT GAT CAT TCA TGG CTC TGT TAT C | 40 mer |
| NE44 (AN4632 3UTR pUC tail) | ACT CTA GAG GAT CCC CGA ATT ACG GTC CGT CTA CGT TG | 38 mer |
| NE45 (AN4632 5UTR nest) | CAC TAG AGC GGA TAC TGA CAT TG | 23 mer |
| NE46 (AN4632 3UTR nest) | GTG TAT CAG TGC TAC GGG ATC | 21 mer |
| NE47 (AN4632 5UTR OUT) | CAA GCG CAA GCG TAT CGA GG | 20 mer |
| NE48 (AN4632 ORF nest FWD) | TCG CTT GGG ATG GTA CGA TTC | 21 mer |
| NE49 (AN4632 ORF nest RVS) | CTT GCT CAA CAG CGT CAA GAC | 21 mer |
| NE50 (AN0164 5UTR pUC tail) | TTC GAG CTC GGT ACC CCG ACT ACT GCA GGT GAG CAG | 36 mer |
| NE51 (AN0164 5UTR pyrG tail) | GAG CAT TGT TTG AGG CGC CGA GAG AAT GGT GGT ACA G | 37 mer |
| NE52 (AN0164 3UTR pyrG/pyroA tail) | GCC TCC TCT CAG ACA GCG CCG CTT TAT GTT TCC TTT GAC | 39 mer |
| NE53 (AN0164 3UTR pUC tail) | ACT CTA GAG GAT CCC CCC ATT AAC TAA CGC CAC TCT CAC | 39 mer |
| NE54 (AN0164 5UTR nest) | TGA AGC GAA CAT AGC TGC CTC | 21 mer |
| NE55 (AN0164 3UTR nest) | GAC GAA TCC CTC AAC CGT TGG | 21 mer |
| NE56 (AN0164 5UTR OUT) | CAA CAG ATC CTG CTC TTA GGA G | 22 mer |
| NE57 (AN0164 ORF nest FWD) | CAT CGA CCT GGA CGA GTG C | 19 mer |
| NE58 (AN0164 ORF nest RVS) | GTA GTC GAT CAC AGG ACC CTG | 21 mer |
| NE59 (AN4632 5UTR pyroA tail) | CCA GCA TCT GAT GTC CGG TGT TAT GGA TGC TCG ACG C | 37 mer |
| NE60 (AN0164 5UTR pyroA tail) | CCA GCA TCT GAT GTC CGC CGA GAG AAT GGT GGT ACA G | 37 mer |
| NE61 (AN6190 ORF linker tail) | CAC CGC TAC CAC CTC CGT CTT TTT TGG CAG GCT CCT CTT | 39 mer |
| NE62 (AN6190 3UTR pyrG/pyroA tail) | GCC TCC TCT CAG ACA GTT GCT GAC ATG TGG AAA CCA AAT TAT C | 43 mer |
| NE63 (AN1010 ORF linker tail) | CAC CGC TAC CAC CTC CCT CAG AGC CTC CCG GAA CAT | 36 mer |
| NE64 (AN1010 3UTR pyrG/pyroA tail) | GCC TCC TCT CAG ACA GTG TAG CGC ACG CTG CTG ATA C | 37 mer |
| NE65 (AN6611 ORF linker tail) | CAC CGC TAC CAC CTC CTG CCC CCT CCA CCT GTA GC | 35 mer |
| NE66 (AN6611 3UTR pyrG/pyroA tail) | GCC TCC TCT CAG ACA GTA GAT CTG AAA TAT CTC GCA TAT GAT G | 43 mer |
| NE67 (AN4632 ORF linker tail) | CAC CGC TAC CAC CTC CCT TAT CCA TTT TCT GCC ACC CGT | 39 mer |
| NE68 (AN4632 3UTR pyrG/pyroA tail) | GCC TCC TCT CAG ACA GCT GAT CAT TCA TGG CTC TGT TAT C | 40 mer |
| NE69 (AN0164 ORF linker tail) | CAC CGC TAC CAC CTC CCA GAA AGT AGT CGA TCA CAG GAC | 39 mer |
| NE70 (AN0164 3UTR pyrG/pyroA tail) | GCC TCC TCT CAG ACA GTA CCC TTT TTC ATT CGT ATT TCG CC | 41 mer |
| NE71 (AN0164 3UTR pUC tail) | ACT CTA GAG GAT CCC CTA CCT GCT GAA TCC AGA CAG TAC | 39 mer |
| NE84 (5UTR AN6190 pUC tail) | AGC TCG GTA CCC ATT TGA ATT CTC TCT TGG GTG ATC TAG A | 24 mer |
| NE85 (3UTR AN6190 pUC tai) | CAT CTG ATG TCC ATT TAG TCC TAT CCA CAC TCC CTA AC | 22 mer |
| NE87 ( 3UTR AN1010 pUC tail) | ATC TGA TGT CCA TTT TCG ACC TTA GCT CTG GTG CAC | 20 mer |
| NE86 (5UTR AN1010 pUC tail) | AGC TCG GTA CCC ATT TGC CAA CTA CCG TGA GGG GA | 19 mer |
| NE88 (5UTR AN6611 pUC tail) | CTC GGT ACC CAT TTA ACC TCT TTC TCA CAA CCG ACA ATG | 23 mer |
| NE89 (3UTR AN6611 pUC tail) | GCA TCT GAT GTC CAT TTA CGT TGT TAG AGG TTG CGA TCA C | 22 mer |
| NE90 (AN4632 5UTR pUC tail) | GAG CTC GGT ACC CAT TTG TGG AGA TGC GGG GAT GGC | 19 mer |
| NE91 (AN4632 3UTR pUC tail) | CAT CTG ATG TCC ATT TGT GAA GAC CGT GAG TTG CTT GAT | 23 mer |
| NE92 (AN0164 5UTR pUC tail) | AGC TCG GTA CCC ATT TCT CAC ATC TGG CGT CTG AAG C | 21 mer |
| NE93 (AN0164 3UTR pUC tail) | CAT CTG ATG TCC ATT TTC CAT AAT GGT TCT CTT GCA CAG C | 24 mer |
| NE94 strA(5UTR AN8071 pUC tail) | AGC TCG GTA CCC ATT TGT TCT AGA AGA GTG GCC ACA AG | 22 mer |
| NE95 strA(3UTR AN8071 pUC tail) | CGC AGT AGC CGT AAC AGC AG | 20 mer |
| OZG1110 (*strA* ptrA 5) | ACA ATG CGA AGC TGA AGC GAC | 21 mer |
| OZG1111 (*strA* pyroA 5) | ACA ATG CGA AGC TGA AGC GAC | 21 mer |
| OZG1112 (*strA* ptrA 3) | TAA CGC AGT GGA TGT GGC CT G | 21 mer |
| OZG1113 (*strA* pyroA 3) | CAT CAG TGC CTC CTC TCA GAC | 21 mer |
| OZG1114 (*strA* 3 for mRFP) | CAC CGC TAC CAC CTC CTC TCG AG | 23 mer |
| OZG1115 (*strA* 3 UTR pyrG) | GCC TCC TCT CAG ACA GCG C | 19 mer |
| OZG1025 (*strA* 5 UTR PmeI) | CCC AGG TTC CTT TGC GAT ATA G T | 23 mer |
| OZG1027 (*strA* 3 natR) | CAT GCC CTG CCC CTG ACG C | 19 mer |
| OZG1028 (*strA* 3 UTR PmeI) | ACT CTA GAG GAT CCC CGT TTA AA | 23 mer |
| OZG1026 (*strA* GFP & TAP fuser) | TAC CAC CGC TAC CAC CTC TCG AG | 23 mer |
| OSBRT1 (*laeA* 5UTR) | CAC AAC CAC TAC AGC TAC CAC | 21 mer |
| OSBRT2 (*laeA* 3UTR) | GCA ACC GCG TAT CTG GTC G | 19 mer |
| OSBRT7 (*ipnA* 5UTR) | GAG AGT AGC CCA GCA AAT CG | 20 mer |
| OSBRT8 (*ipnA* 3UTR) | GGC ACG AAT CGC AAG GTC C | 19 mer |
| OSBRT9 (*acvA* 5UTR) | GAC AAG GAC AAC CGT GAT G | 19 mer |
| OSBRT10 (*acvA* 3UTR) | GCA CAC CAT TAC TGC TAG AGG | 21 mer |
| OSBRT11 (*aatA* 5UTR) | CCA TTG ACT TCG CAA CTG GC | 20 mer |
| OSBRT12 (*aatA* 3UTR) | CGT ACG AGT GTT GAG CAT GAC | 21 mer |
| OSBRT13 (*tdiA* 5UTR) | CGA TGC CTG GAG TGC GAA TG | 20 mer |
| OSBRT14 (*tdiA* 3UTR) | GCC GTT GCT GTC AAT GAA CG | 20 mer |
| OSBRT19 (*brlA* 5UTR) | CTC GAA GAC ATG CGA AAT CAG | 21 mer |
| OSBRT20 (*brlA* 3UTR) | CAG GAG TTC GTT CGT ACG TG | 20 mer |
| OSBRT57 (*aflR* 5UTR) | CCT TCG CTT CTT GAG GGT ATG G | 22 mer |
| OSBRT58 (*aflR* 3UTR) | GCA GTA GGA GTG GCT TGT GGT G | 22 mer |
| OSBRT68 (*stcE* 5UTR) | GCA TCT CGA TGT AGT GAT CG | 20 mer |
| OSBRT69 (*stcE* 3UTR) | CTA GTC GCC TGG AAC AGT AG | 20 mer |
| OSBRT70 (*stcQ* 5UTR) | GGT TGT AGC GTC TTT GCA ACG | 21 mer |
| OSBRT71 (*stcQ* 3UTR) | GAA CAT CGT TGC AGA ACG TGG | 21 mer |
| OSBRT76 (*veA* 5UTR) | CGA TCC AGA GCC TCT CAG AG | 20 mer |
| OSBRT77 (*veA* 3UTR) | GGT CAT CAT GAC CGA ACG AC | 20 mer |
| OSBRT78 (*velB* 5UTR) | CCT CCC ACA ATC GGA TAT TGC | 21 mer |
| OSBRT79 (*velB* 3UTR) | GGG ATC TTG ATT CCT TGG TTC | 21 mer |
| OSBRT88 (*abaA* 5UTR) | GCA ACC CGA GTG TAT GGT ATC | 21 mer |
| OSBRT89 (*abaA* 3UTR) | CAC GTT CCC AGA CGT ATT CTG | 21 mer |
| OSBRT116 (*catC* 5UTR) | GCC ACC ATG TTC TGG GAC TAC | 21 mer |
| OSBRT117 (*catC* 3UTR) | GTA GGT GTG ACC GGA GTA GC | 20 mer |
| OSBRT118 (*sodB* 5UTR) | CTA CGT CAA CAG CTA CAA CAC | 21 mer |
| OSBRT119 (*sodB* 3UTR) | GAG AGA GCA CCA GAA GGA G | 19 mer |
| OSBRT120 (*nsdD* 5UTR) | GGC ATT ATG CAA ACA GAG CAC | 21 mer |
| OSBRT121 (*nsdD* 3UTR) | GCA TCA GAG CGT CGT GGT TC | 20 mer |
| BK280 (*benA* 5UTR) | GAT GGC TGC CTC TGA CTT C | 19 mer |
| BK281 (*benA* 3UTR) | GCA TCT GGT CCT CAA CCT C | 19 mer |
| BK295 (*setA* 5UTR) | CAC TCA GGA GCG ACC ATA TC | 20 mer |
| BK296 (*setA* 3UTR) | CTT GCT GAG CCT CGT GAA TG | 20 mer |
| BK546 (mpkA 5UTR 5) | TTC GAG CTC GGT ACC CCA GCA ACT TGA ACA CAA GTA CTG AC | 41 mer |
| BK547 (mpkA 5UTR 3) | CAC CGC TAC CAC CTC CCG CAT CCA TCC CCC GCT G | 34 mer |
| BK548 (mpkA 3UTR 5) | GCC TCC TCT CAG ACA GTT AGT CAA GCG TTG GTA TTT ATT TAT CC | 44 mer |
| BK549 (mpkA 3UTR 3) | ACT CTA GAG GAT CCC CCT GAT CAC CGA CTC CAC CAA G | 37 mer |
| BK550 (mpkA NEST FWD) | CAG CAA CTT GAA CAC AAG TAC TGA C | 25 mer |
| BK551 (mpkA NEST RV) | CTG ATC ACC GAC TCC ACC AAG | 21 mer |
| BK552 (mpkC 5UTR 5) | TTC GAG CTC GGT ACC CCC TTC CCT CAC CAG TAC ACC | 36 mer |
| BK553 (mpkC 5UTR 3) | CAC CGC TAC CAC CTC CCG AAA AGG AAT TGG GGT CCA GC | 38 mer |
| BK554 (mpkC 3UTR 5) | GCC TCC TCT CAG ACA GGG TTG AGT TGA GTC TTT TCC TTT TTC | 42 mer |
| BK555 (mpkC 3UTR 3) | ACT CTA GAG GAT CCC CGC TGA ACT CAT CGA ACT CAT CG | 38 mer |
| BK556 (mpkC NEST FWD) | CCT TCC CTC ACC AGT ACA CC | 20 mer |
| BK557 (mpkC NEST RV) | GCT GAA CTC ATC GAA CTC ATC G | 22 mer |
